# Exploration of effective biomarkers and infiltrating immune cells in metastatic colorectal cancer based on bioinformatics analysis

**DOI:** 10.1038/s41598-025-18589-4

**Published:** 2025-09-26

**Authors:** Junming Huang, Yunhan Guo, Xiaoying Lv, Yaohang Long, Xianyi Wang, Dawei Jin, Hongmei Liu

**Affiliations:** 1 School of Public Health, The Key Laboratory of Environmental Pollution Monitoring and Disease Control, Ministry of Education, Guizhou Medical University, Guiyang, 561113 China; 2 Engineering Research Center of Health Medicine Biotechnology of Institution of Higher Education of Guizhou Province, School of Biology and Engineering, Guizhou Medical University, Guiyang, 561113 China; 3 School of Clinical Medicine, Guizhou Medical University, Guiyang, 561113 China; 4 Key Laboratory of Biology and Medical Engineering, Immune Cells and Antibody Engineering Research Center of Guizhou Province, School of Biology and Engineering, Guizhou Medical University, Guiyang, 561113 China; 5 School of Basic Medicine Science, Guizhou Medical University, Guiyang, 561113 China; 6 Laboratory Animal Center of Guizhou Medical University, Guiyang, 561113 China; 7 Research Institute of Guizhou Huada Life Big Data Guiyang, Guizhou, 550025 China

**Keywords:** Metastatic colorectal cancer, Infiltrating immune cells, Differentially expressed genes, Bioinformatics analysis, Tumor microenvironment, Gastrointestinal cancer, Tumour biomarkers, Tumour immunology

## Abstract

Colorectal cancer (CRC) ranks as the third most prevalent malignancy globally and represents the second leading cause of cancer-related mortality worldwide. Metastatic colorectal cancer (mCRC) is clinically classified as an advanced-stage malignancy, characterized by therapeutic resistance and substantially diminished survival outcomes. The 5-year survival rate for mCRC is significantly lower than that for early-stage CRC. A multi-dimensional computational framework was implemented to dissect CRC transcriptomics. Gene expression profiles were systematically acquired from TCGA and GEO repositories. A series of data were then analyzed using ssGSEA algorithm, xCell algorithm, edgeR, limma, DAVID enrichment analysis, CytoHubba, ROC logistic regression and correlation analysis. Immune cell infiltration analysis revealed 7 tumor-infiltrating immune cell subtypes exhibiting significant abundance disparities between metastatic and non-metastatic colorectal cancer cohorts. Further integrative analysis identified 28 immune-related metastatic colorectal cancer differentially expressed genes (ICDEGs) in metastatic lesions. Through comprehensive analysis, 9 pivotal hub genes (*AGTR1*, *CD86*, *CMKLR1*, *FGF1*, *FYN*, *IL10RA*, *INHBA*, *TNFSF13B*, and *VEGFC*) were successfully identified. Notably, *AGTR1*, *CD86*, *CMKLR1* and *TNFSF13B* genes have been rarely reported in mCRC. Furthermore, our correlation studies revealed significant inverse relationships between epithelial cells and three specific genes: *TNFSF13B*, *CD86*, and *IL10RA*. The identified 9 hub genes demonstrate significant potential as reliable diagnostic biomarkers for mCRC. Moreover, these molecular markers may contribute to disease pathogenesis through their dynamic interactions with tumor-infiltrating immune cells, suggesting a crucial role in the tumor microenvironment.

**Supplementary Information:**

The online version contains supplementary material available at 10.1038/s41598-025-18589-4.

## Introduction

CRC, a prevalent malignant neoplasm of the gastrointestinal tract, ranks as the third most frequently diagnosed cancer worldwide and represents the second leading cause of cancer-related mortality^1^. Multiple risk factors, including demographic aging, Westernized dietary patterns prevalent in high-income nations, increasing obesity rates, and sedentary lifestyles, have been identified as significant contributors to elevated CRC incidence^2^. Epidemiological data indicate that among newly diagnosed CRC cases, approximately 20% present with metastatic disease at initial diagnosis, while an additional 25% subsequently develop metastatic progression after localized disease manifestation^3^. Surgical intervention remains the sole curative approach for localized CRC, with adjuvant chemotherapy typically indicated for cases demonstrating lymph node involvement. While multimodal therapy incorporating surgery, radiotherapy, and chemotherapy constitutes the fundamental treatment paradigm for rectal cancer, systemic chemotherapy remains the cornerstone of therapeutic strategies for advanced-stage CRC^4^. Despite comprehensive therapeutic interventions, including surgical resection of colorectal tumors, mCRC exhibits a substantial propensity for disease recurrence^5^.

The initial clinical manifestations of CRC are frequently subtle and challenging to identify, while metastatic progression is characterized by aggressive disease advancement. Early detection and intervention have been demonstrated to substantially enhance survival outcomes and significantly improve prognostic indicators in CRC patients^6^. The accelerated advancement and extensive implementation of microarray sequencing platforms^7^ and RNA sequencing-based transcriptomic profiling^8^ have significantly enhanced the application of bioinformatics in novel biomarker discovery across various diseases. These technological innovations facilitate not only the investigation of disease-associated epigenetic modifications but also provide critical insights into fundamental molecular pathways and pathogenic mechanisms^9^. Utilizing integrated data from the Gene Expression Omnibus (GEO) and The Cancer Genome Atlas (TCGA) databases, Chen et al. successfully identified novel gene signatures with diagnostic and prognostic potential for CRC. Their comprehensive bioinformatics approach enabled the identification of conserved differentially expressed genes (DEGs) across CRC samples, establishment of an extensive protein-protein interaction (PPI) network, and subsequent prioritization of the ten most significant hub genes through systematic analysis^10^. Recent studies have demonstrated that within the TGF-β pathway in CRC, *LINC01705* expression is significantly correlated with and co-upregulated alongside the *SERPINE1* gene^11^while *LINC01614* has been identified as a novel lncRNA significantly associated with the *SPP1* gene^12^. Both studies employed TCGA data mining combined with experimental validation, highlighting the importance of these lncRNA-gene interaction axes in CRC pathogenesis and their value as potential therapeutic targets and diagnostic biomarkers^11,12^. In CRC, significant alterations in the expression of the membrane transporters *ANO7* and *SLC38A4* have been identified. These findings, integrating differential gene analysis and feature selection of the TCGA-COAD database with multi-platform validation, confirmed their dysregulated expression patterns in CRC tissues and proposed their potential as prognostic biomarkers for colorectal cancer^13^. In our preceding investigation, Lv and colleagues employed regression modeling to establish quantitative relationships between messenger RNA (mRNA) expression profiles and their corresponding regulatory transcription factors (TFs). The study introduced the novel concept of “dark biomarkers,” defined as mRNA genes that, while not exhibiting differential expression in metastatic colon cancer (mCC), demonstrated significant correlations with mqTrans values in mCC pathogenesis^14^.

Inflammatory cells significantly contribute to both tumor initiation and progression, while adaptive immune mechanisms are crucial in controlling neoplastic expansion and metastasis. The development and advancement of CRC are fundamentally regulated by complex tumor-host interactions within the specialized tumor microenvironment. Recent research has progressively elucidated the multifaceted roles of immune cells in CRC pathogenesis, with particular scientific focus on their involvement in tumor promotion, immunological surveillance, and the intricate dynamics of immune evasion mechanisms^15^. Single-cell RNA sequencing analyses of colorectal cancer specimens have revealed a substantial expansion of tumor-associated suppressor cells derived from both macrophage and granulocyte myeloid lineages. These cells demonstrate markedly enhanced immunosuppressive characteristics compared to their counterparts in normal tissue, representing the most potent immunosuppressive population within the tumor immune microenvironment^16^. Given these findings, the establishment of a comprehensive framework for evaluating immune cell functionality in mCRC and identifying immune-related diagnostic markers has become imperative. Such an approach would facilitate a deeper understanding of the immunological underpinnings of mCRC, potentially leading to transformative advances in diagnostic precision, therapeutic interventions, and the development of innovative immunotherapeutic approaches. The xCell algorithm represents a sophisticated gene signature-based methodology that enables comprehensive cell type enrichment analysis through the examination of gene expression profiles across 64 distinct immune and stromal cell populations. This computational approach has been extensively validated through extensive computational simulations and empirical immunophenotyping studies^17^. Consequently, this methodology facilitates a comprehensive and systematic evaluation of immune cell infiltration patterns within CRC tissues, serving as a crucial analytical tool for deciphering the complex immune microenvironment associated with colorectal carcinogenesis.

This investigation marks the first application of the xCell algorithm to comparatively analyze immune infiltration patterns between CRC and mCRC. Subsequently, we implemented an integrated bioinformatics pipeline to identify and characterize DEGs associated with CRC progression to metastasis. Our analytical framework incorporated differential gene expression profiling, Gene Ontology (GO)^18^ and Kyoto Encyclopedia of Genes and Genomes (KEGG)^19^ pathway enrichment analyses, PPI network construction, hub gene identification, and ROC-based logistic regression modeling to elucidate the pathological mechanisms and immune landscape of mCRC. The final analytical phase employed Spearman’s correlation analysis^20^ to investigate potential associations between identified biomarker candidates and significantly infiltrating immune cell populations, providing insights into their potential interplay within the tumor microenvironment. Collectively, this investigation contributes to the identification of novel diagnostic biomarkers for mCRC while offering valuable insights that could enhance diagnostic precision and inform the development of immunotherapeutic strategies, ultimately advancing prevention, detection, and therapeutic approaches for metastatic colorectal cancer.

## Methods and materials

### Data acquisition and processing

**Fig. 1:**
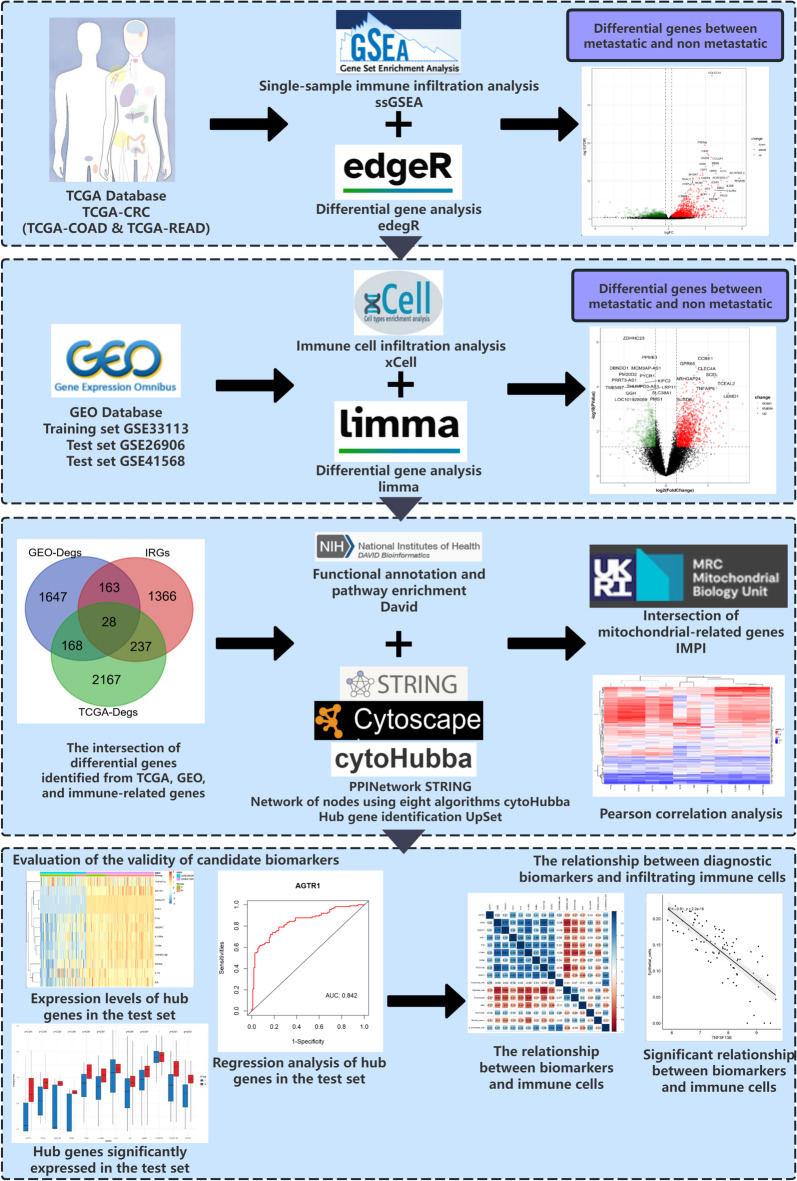
Methodological workflow of the study. The investigation involved systematic screening of TCGA-CRC and GEO databases to identify immune-related differentially expressed genes. Subsequent analyses included immune cell infiltration profiling and functional enrichment analysis to identify Hub genes, followed by exploration of their correlation with mitochondrial-associated genes and validation of Hub gene functionality.

A schematic representation of the methodological workflow implemented in this investigation is presented in Fig. 1. Clinical data corresponding to colon adenocarcinoma (TCGA-COAD) and rectal adenocarcinoma (TCGA-READ) were retrieved through the R/Bioconductor package “TCGAbiolinks“^21^a specialized tool designed for integrative analysis of TCGA genomic and clinical datasets. Transcriptomic analysis was performed on 567 CRC patients with complete clinical information. Gene expression profiles associated with mCRC were acquired from the GEO database (https://www.ncbi.nlm.nih.gov/geo/)^22^, comprising three transcriptomic datasets (Table 1). The GSE33113 dataset^23^ served as the training cohort, while GSE26906^24^ and GSE41568^25^ functioned as independent validation sets. Data acquisition from the GEO repository was performed using the R package “GEOquery“^26^followed by sequence matrix probe re-annotation utilizing the “AnnoProbe” R package for enhanced genomic annotation accuracy. Metastatic status annotations were systematically curated according to the TNM classification system, with the M category specifically employed to determine the presence or absence of distant metastasis^27^. Samples were classified as mCRC when exhibiting an M stage > 0, documented metastatic progression, or histopathological evidence of distant organ involvement, while all other cases were designated as non-metastatic CRC. A comprehensive collection of immunity-associated genes was acquired from the ImmPort repository (http://www.immport.org)^28^, with the obtained immune-related gene set being designated as IRGs.

**Table 1:** Characteristics of the four datasets utilized in this investigation. Three GEO datasets were profiled using the affymetrix genechip human genome U133 plus 2.0 array (platform GPL570). The “summary” column delineates the distribution of primary (P) and metastatic (M) cancer specimens across the datasets.

Datasets	Platform	Species	Samples	Summary	Age (median, range)	Male/female	Metastasis Sites	TNM stage
TCGA-CRC	TCGA	Homo sapiens	567	477P vs. 90 M	68 (31–90)	296/271	None	Stage I: 78 Stage II: 161 Stage III: 232 Stage IV: 87
GSE33113	GPL570	Homo sapiens	89	71P vs. 18 M	73 (34–95)	41/49	None	None
GSE26906	GPL570	Homo sapiens	90	69P vs. 21 M	69 (31–94)	43/47	Liver (77.27%), Lung (13.63%), Bone (9.09%)	None
GSE41568	GPL570	Homo sapiens	133	39P vs. 94 M	None	None	Liver (81.91%), Lung (8.51%), Other (6.38%)	None

### Screening for DEGs associated with immune genes

Data preprocessing and normalization were performed using the R package “edgeR“^29^followed by implementation of a negative binomial generalized log-linear model^30^ to identify differentially expressed genes (TCGA-DEGs) between metastatic and non-metastatic patients within the TCGA-CRC cohort. Particular emphasis was placed on genes exhibiting subtle but potentially biologically relevant expression differences. This investigation prioritized genes demonstrating subtle yet biologically significant expression variations with potential marker utility. To control the false positives in multiple testing, Benjamini-Hochberg false discovery rate (FDR) correction was performed on the p-values for all genes. Consequently, we established more inclusive selection criteria (|log_2_Fold Change| ≥ 0.25 and *p* < 0.05) to capture a broader spectrum of differentially expressed genes with potential biological relevance.

The R package “limma“^31^ was subsequently employed for normalization and analysis of microarray-based transcriptomic data from the GEO repository, enabling identification of differentially expressed genes (GEO-DEGs) between metastatic and non-metastatic cohorts using the established thresholds of |log_2_Fold Change| ≥ 0.25 and *p* < 0.05. Genes demonstrating overlap among TCGA-DEGs, GEO-DEGs, and IRGs were designated as immune-related metastatic colorectal cancer differentially expressed genes (ICDEGs). The quantification of ICDEGs was accomplished through Venn diagram analysis utilizing the interactive web-based tool Draw Venn Diagram (http://bioinformatics.psb.ugent.be/webtools/Venn/). Gene expression patterns of TCGA-DEGs, GEO-DEGs, and ICDEGs were graphically represented through volcano plots generated using the R package “ggplot2”, while the expression profile of ICDEGs was additionally visualized as a heatmap employing the “pheatmap” package.

### Estimation of immune cell infiltration

To characterize the immune landscape, we quantified the relative abundance of 28 functionally distinct immune and stromal cell populations defined by Bindea et al.^32^ This curated gene signature set encompasses key players in anti-tumor immunity, immunosuppression, antigen presentation, and inflammation, all of which have established roles in CRC progression and metastasis^15,16,32^. We evaluated immune cell infiltration patterns within TCGA-CRC expression profiles, thereby elucidating features of the tumor immune microenvironment. The R packages “GSEABase” and “GSVA“^33^ were employed to conduct single-sample gene set enrichment analysis (ssGSEA) on TCGA-CRC datasets. The ssGSEA approach was subsequently applied to evaluate immune cell infiltration patterns in tissue specimens^34^. Visualization of immune infiltration profiles was achieved using the “ggplot2” R package^35^.

To obtain a more comprehensive and granular view of the tumor microenvironment (TME), including both immune and non-immune stromal components critically involved in CRC metastasis, we additionally employed the xCell algorithm^17^.The xCell algorithm (https://xcell.ucsf.edu/) represents a gene signature-based computational method that generates relative infiltration scores for 64 distinct immune and stromal cell populations derived from microarray-based gene expression profiles. This investigation employed the xCell algorithm to characterize and compare immune cell infiltration patterns between mCRC and CRC tissues. Principal component analysis (PCA) was subsequently implemented to statistically validate the significance of observed inter-group differences in immune infiltration profiles^36^. Immune cell populations demonstrating significant infiltration were subsequently subjected to correlation analysis using Spearman’s rank correlation method. Benjamini-Hochberg FDR correction was applied. Immune cells with significantly differential infiltration were defined as those with FDR-corrected *p* < 0.01.

### Functional enrichment analysis

Functional characterization of ICDEGs was conducted through Gene Ontology (GO) analysis encompassing Biological Processes (BP), Cellular Components (CC), and Molecular Functions (MF). Concurrently, KEGG pathway analysis was employed to elucidate the associated signaling pathways and higher-order biological functions of ICDEGs. Both GO and KEGG annotations and analyses were executed using the DAVID bioinformatics resource (https://david.ncifcrf.gov/)^37^. Significantly enriched GO terms and KEGG pathways were defined as those with FDR-adjusted *p* < 0.05. The top 15 statistically significant pathways meeting this threshold are reported. The top 15 statistically significant pathways from both analyses were graphically represented as bubble plots utilizing the R package “clusterProfiler“^38^. Functional enrichment analysis with chord diagram visualization were performed through the SangerBox^39^ to elucidate the potential biological roles of ICDEGs.

### Identification of hub genes

The PPI network of ICDEGs was established using the STRING database (https://cn.string-db.org)^40^, with interactions exceeding a confidence score threshold of 0.4 being retained for subsequent analysis of ICDEG interrelationships. The network was subsequently constructed and visualized using Cytoscape software (version 3.10.1)^41^. Hub gene identification was performed by applying eight topological analysis algorithms from the CytoHubba plugin^42^: Maximum Clique Centrality (MCC), Degree, Edge Percolated Component (EPC), Bottleneck, Eccentricity, Closeness, Radiality, and Betweenness, with the top 15 nodes (genes) prioritized based on their composite scores. Hub genes were identified through the intersection of eight algorithmic predictions using the SangerBox platform’s R package “UpSet“^43^with subsequent validation performed against independent gene expression datasets (GSE26906 and GSE41568).

### Identification of hub gene correlations with mitochondrial function-related genes

The Mitochondrial Protein Index (IMPI; https://mitominer.mrc-mbu.cam.ac.uk/) was retrieved from an established online repository^44^. The intersection between GEO-DEGs and mitochondrial function-related genes (MFRGs) was identified and designated as differentially expressed mitochondrial function-related genes (DE-MFRGs). Pairwise Pearson correlation analyses between Hub genes and DE-MFRGs were conducted utilizing the R package “correlation”, with the resultant correlation matrices visualized as comprehensive heatmaps.

### Assessment of the diagnostic validity of candidate biomarkers

ROC analysis of logistic regression models was conducted using the R package “pROC“^45^. The area under the ROC curve (AUC) served as the primary metric to assess the diagnostic efficacy of candidate biomarkers for mCRC within the transcriptomic profiles of the GSE26906 and GSE41568 datasets.

### Survival analysis of diagnostic biomarkers

To validate the clinical prognostic value of candidate diagnostic biomarkers, survival analysis was performed for the identified Hub genes using the GEPIA (Gene Expression Profiling Interactive Analysis, http://gepia.cancer-pku.cn) online platform^46^. The analysis was based the TCGA-CRC dataset, which includes transcriptomic expression profiles and clinical overall survival (OS) data. Utilizing the “Survival Analysis” module in GEPIA, the median expression cutoff was applied to automatically stratify the TCGA-CRC cohort into high-expression and low-expression groups. The log-rank test was employed to compare survival curve differences between the two groups. OS curves were plotted using months as the time scale. Core diagnostic biomarkers identified in previous screening were individually input into the GEPIA system to generate corresponding Kaplan-Meier (KM) survival curves.

### Relationship between diagnostic biomarkers and infiltrating immune cells

To elucidate the potential involvement of immune cell infiltration in mCRC pathogenesis, Spearman correlation analyses were performed between candidate diagnostic biomarkers and infiltrating immune cell populations using the R package “correlation”. Benjamini-Hochberg FDR correction was applied for multiple testing. A statistically significant correlation was defined as |*r*| > 0.70 with an FDR-corrected *p* < 0.05^47^. The resultant associations were graphically represented using “corrplot” and “ggplot2” R packages.

## Results

### ICDEGs between mCRC and CRC tissues

**Fig. 2:**
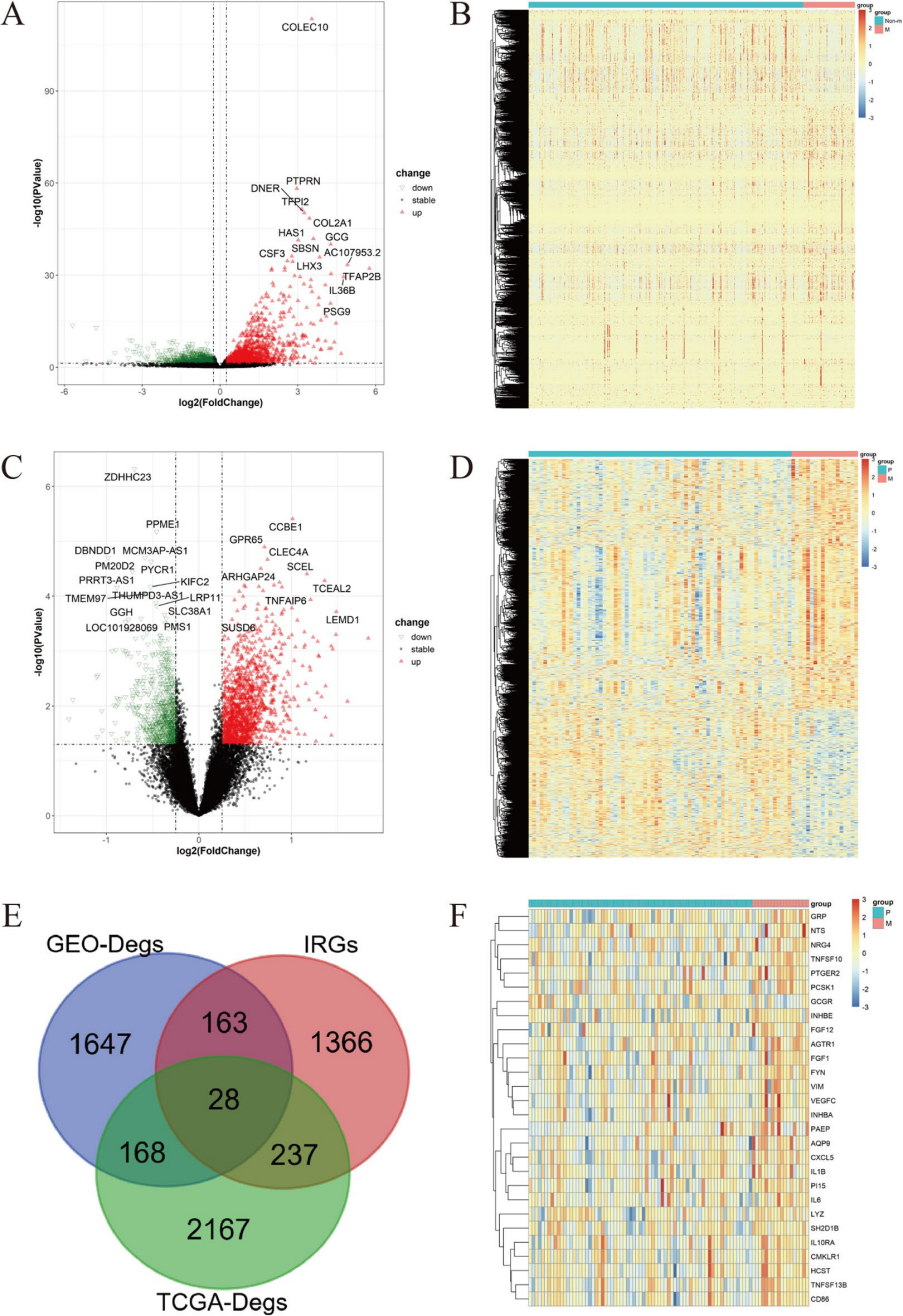
Identification of mCRC-associated DEGs and ICDEGs. (**A**) Volcano plot illustrating TCGA-DEGs; (**B**) Heatmap representation of TCGA-DEGs expression profiles; (**C**) Volcano plot illustrating GEO-DEGs; (**D**) Heatmap representation of GEO-DEGs expression profiles; (**E**) Venn diagram demonstrating the intersection of TCGA-DEGs, GEODEGs, and IRGs; (**F**) Heatmap displaying expression profiles of 28 ICDEGs.

Analysis of the TCGA-CRC dataset, comprising primary and metastatic CRC samples (*P* = 477, M = 90), revealed 2599 TCGA-DEGs (Fig. 2A, B).

Analysis of the training cohort GSE33113, encompassing primary and metastatic samples (*P* = 71, M = 18), identified 2005 GEO-DEGs. Among these, 1254 genes exhibited upregulation while 751 showed downregulation in mCRC specimens relative to their primary counterparts (Fig. 2C, D). Concurrently, 1794 IRGs were curated from established databases. Intersection analysis of 2599 TCGA-DEGs, 2005 GEO-DEGs, and 1794 IRGs yielded a set of ICDEGs (Fig. 2E, F). This integrative approach identified 28 ICDEGs, comprising 27 upregulated and 1 downregulated genes (Table 2).

**Table 2:** ICDEGs between mCRC group and CRC group.

ICDEGs	Gene name	Number
Down-regulated	GCGR	1
Up-regulated	AQP9, PTGER2, FYN, FGF1, PI15, IL10RA, VEGFC, AGTR1, CXCL5, TNFSF13B, CD86, FGF12, INHBE, CMKLR1, HCST, VIM, IL1B, INHBA, IL6, PAEP, GRP, LYZ, TNFSF10, PCSK1, NTS, SH2D1B, NRG4	27

ICDEGs were set as |log_2_Fold Change| ≥ 0.25 and *p* < 0.05.

ICDEGs: immune-related and mCRC-related differentially expressed genes.

### Immune cell infiltration between mCRC and CRC

**Fig. 3:**
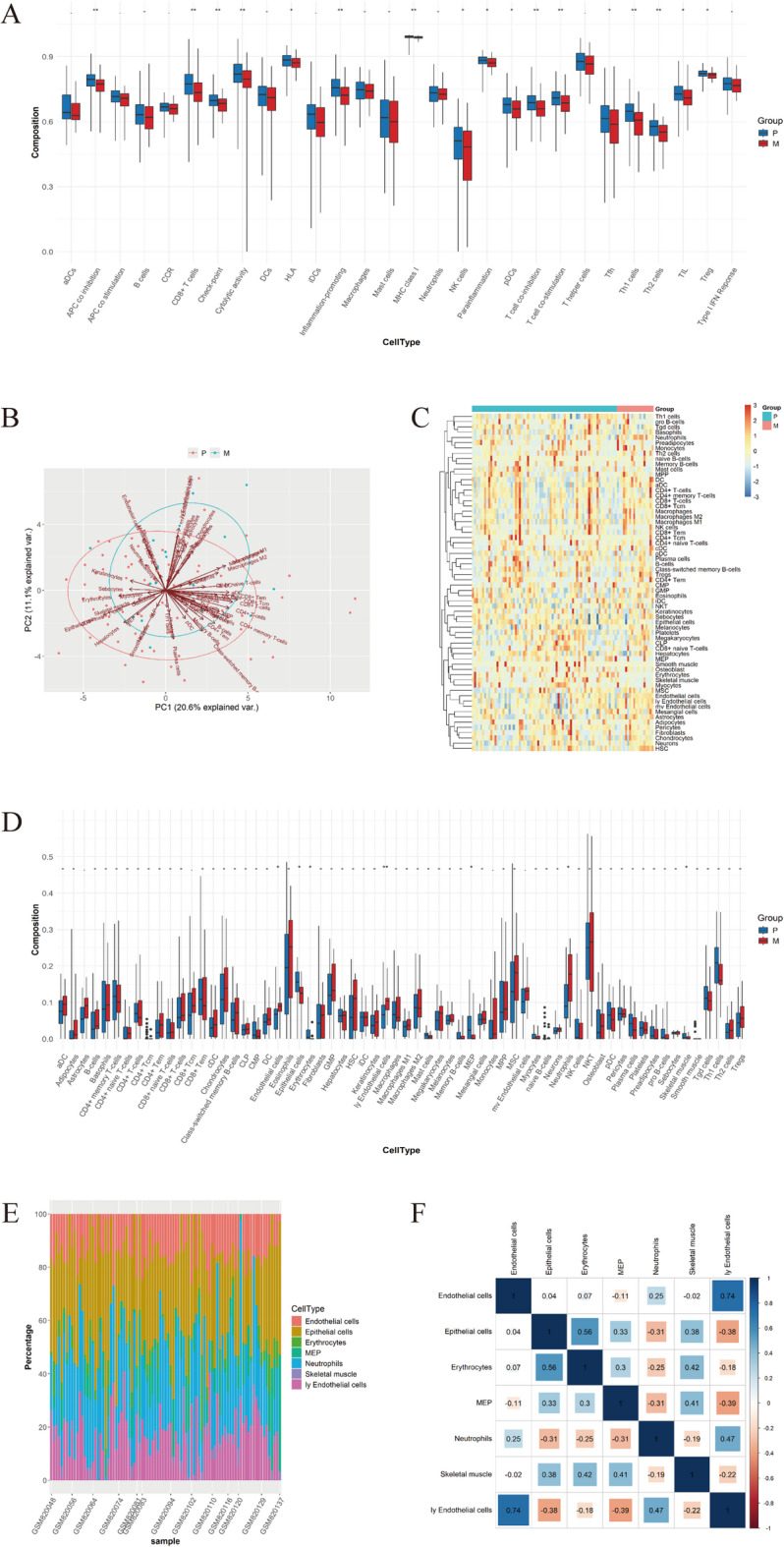
Comparative analysis of immune cell infiltration in mCRC versus CRC. (**A**) Boxplots illustrating specific infiltration patterns of 28 immune cell populations analyzed by ssGSEA algorithm in the TCGA-CRC dataset; (**B**) PCA of 64 immune cell types in the GSE33113 dataset, with corresponding abundance quantification using the xCell algorithm; (**C**) Heatmap visualization of 64 immune cell types in the GSE33113 dataset; (**D**) Boxplot representation of 64 immune cell types in the GSE33113 dataset; (**E**) Relative proportions of 7 distinct infiltrating immune cell types (X-axis: 71 CRC and 18 mCRC samples from the GSE33113 training set; Y-axis: relative percentages of 7 immune cell types per sample; color-coded legend indicates immune cell types); (**F**) Correlation matrix of 7 distinct infiltrating immune cell types in the GSE33113 dataset.

Initial comparative analysis of immune infiltration between CRC and mCRC tissues was conducted using TCGA datasets. The ssGSEA algorithm quantified the infiltration patterns of 28 distinct immune cell populations, with the results visualized as boxplots (Fig. 3A). The analysis revealed that the most prominently distinct immune cell populations between CRC and mCRC tissues predominantly included APC co-inhibition, CD8^+^ T cells, checkpoint molecules, cytolytic activity markers, inflammation-promoting factors, MHC class I molecules, T cell co-inhibition markers, T cell co-stimulation factors, T follicular helper (Tfh) cells, and T helper 1 (Th1) cells.

**Fig. 4:**
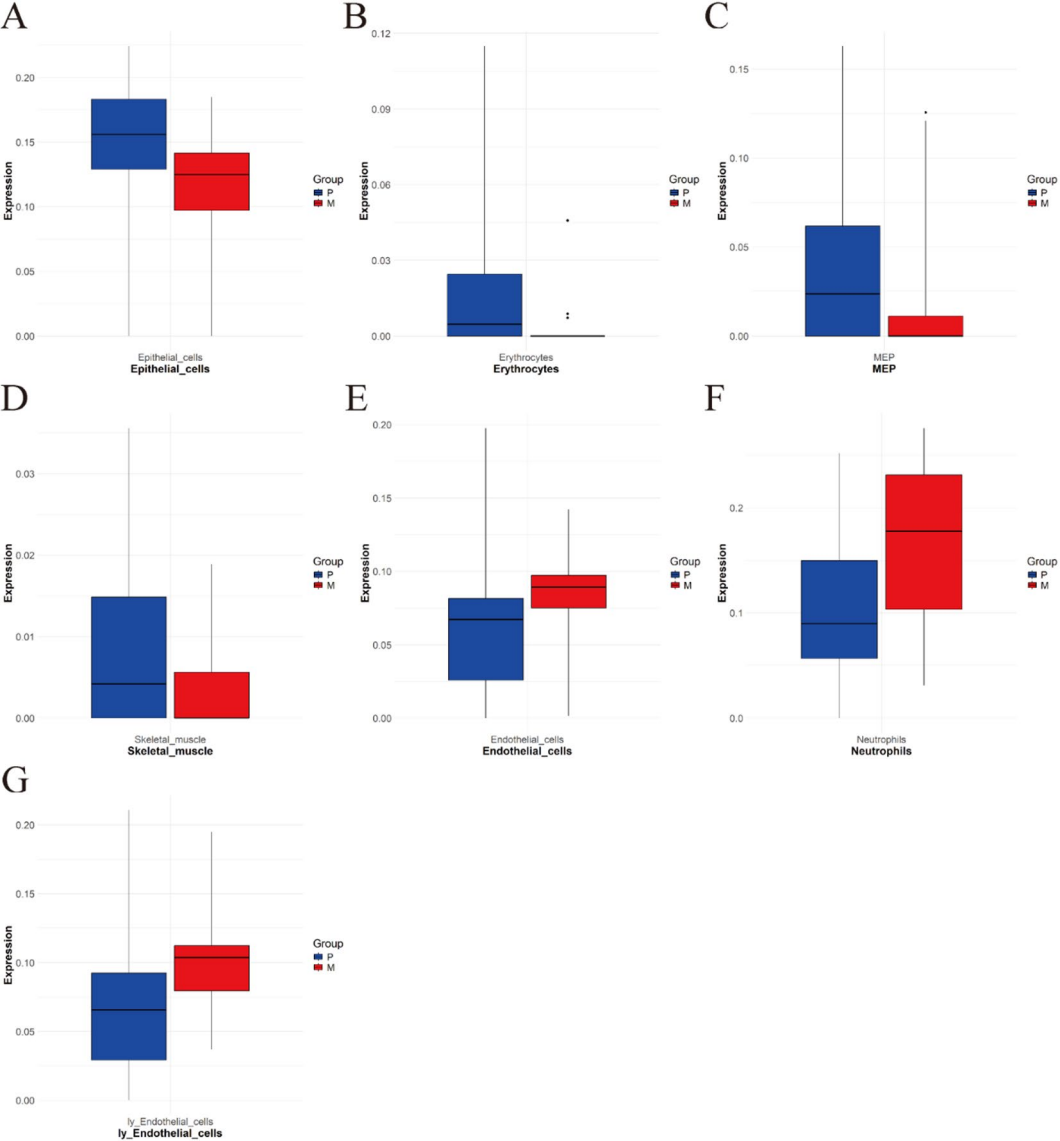
Differential infiltration patterns of immune cells in mCRC. Red coloration denotes expression levels in mCRC, while blue represents CRC expression. The downregulated infiltrating immune cell populations in mCRC comprised (**A**) Epithelial cells; (**B**) Erythrocytes; (**C**) MEP; (**D**) Skeletal muscle, upregulated infiltrating immune cells included (**E**) Endothelial cells; (**F**) Neutrophils; (**G**) ly Endothelial cells.

Subsequent analysis of immune infiltration patterns between CRC and mCRC tissues was performed using GEO datasets. PCA demonstrated statistically significant distinctions in immune cell infiltration profiles between these two tissue types (Fig. 3B). The xCell algorithm was implemented to characterize the specific infiltration patterns of 64 immune cell populations, with the analytical outcomes visualized through heatmaps (Fig. 3C) and boxplots (Fig. 3D). The analysis revealed that the predominant infiltrating immune cell populations in CRC and mCRC tissues primarily consisted of endothelial cells, epithelial cells, erythrocytes, megakaryocyte-erythroid progenitors (MEP), neutrophils, skeletal muscle cells, and lymphatic endothelial cells. Quantitative assessment of the relative proportions of these seven immune cell populations was performed (Fig. 3E). Correlation analysis demonstrated a strong positive association between lymphatic endothelial cells and endothelial cells (*r* = 0.74), while revealing a significant negative correlation between lymphatic endothelial cells and MEP (*r* = -0.39) (Fig. 3F). Comparative analysis revealed a relative reduction in epithelial cells, erythrocytes, MEP, and skeletal muscle cells in mCRC tissues compared to their CRC counterparts (Fig. 4A-D). Conversely, endothelial cells, neutrophils, and lymphatic endothelial cells exhibited significant enrichment in mCRC tissues (Fig. 4E-G).

### GO and KEGG pathway analysis of ICDEGs

**Fig. 5:**
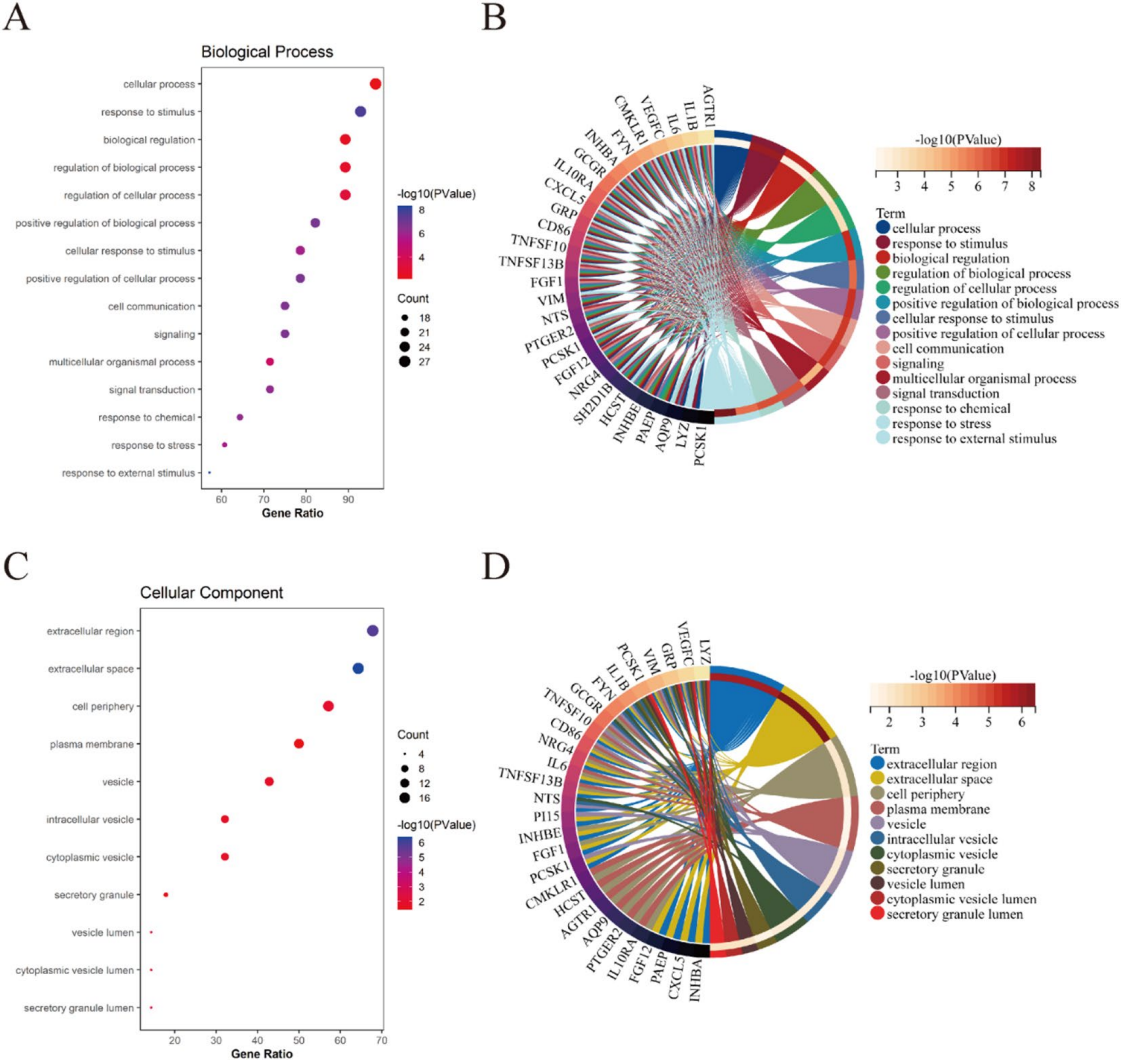
GO analysis of ICDEGs in BP and CC. (**A**, **B**) Bubble plot and circular visualization of GO terms associated with ICDEGs in BP; (**C**, **D**) Bubble plot and circular representation of GO terms related to ICDEGs in CC.

**Fig. 6:**
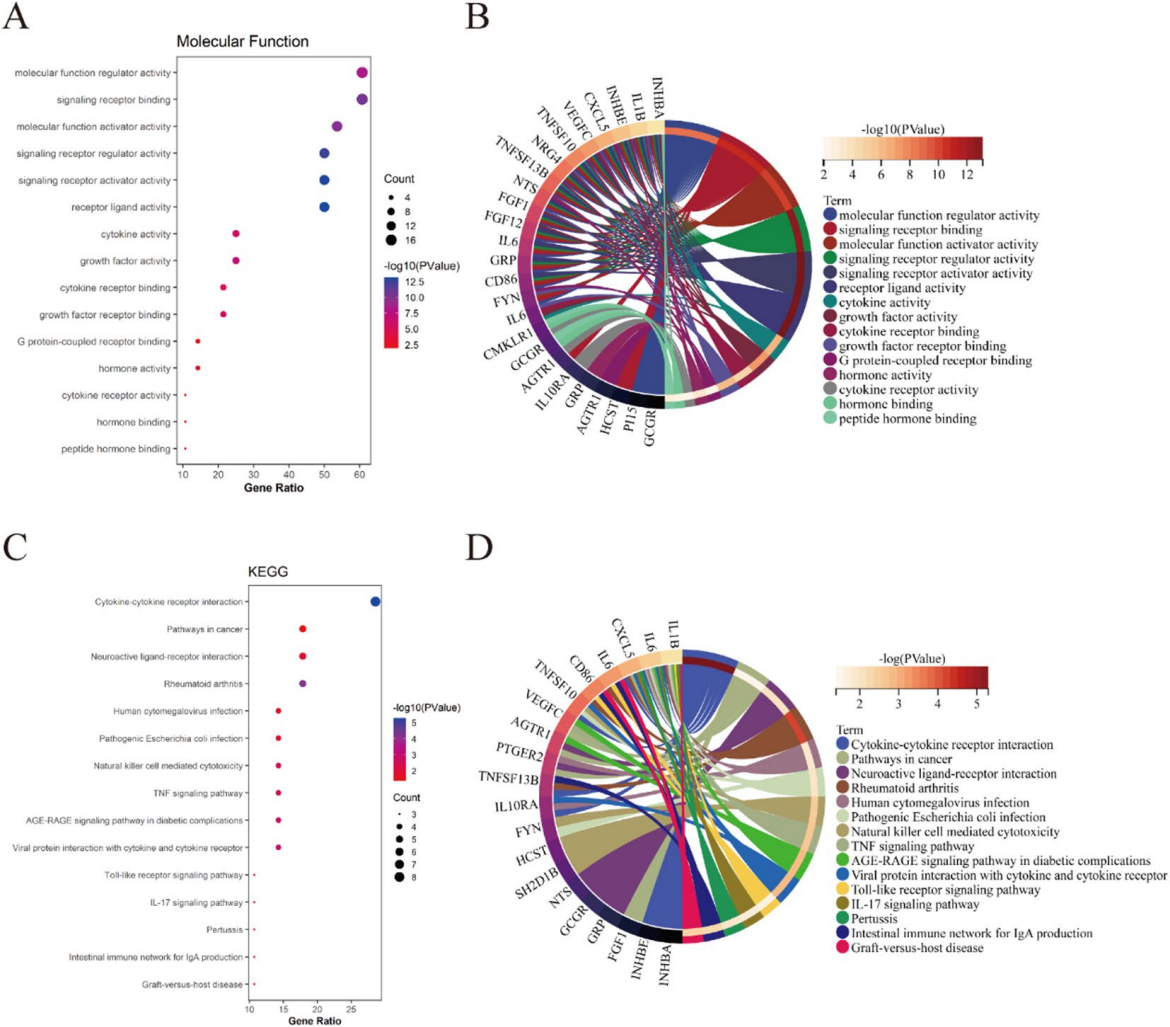
Functional characterization of ICDEGs through MF and KEGG pathway^48,49^ analysis. (**A**, **B**) Bubble plot and circular visualization of GO terms associated with ICDEGs in MF; (**C**, **D**) Bubble plot and circular representation of KEGG pathways related to ICDEGs.

Functional characterization of ICDEGs was performed through GO analysis across three domains: BP, CC, and MF. Within BP, ICDEGs demonstrated significant enrichment in cellular processes, response to stimulus, biological regulation, regulation of biological processes, and regulation of cellular processes (Fig. 5A, B). For CC, ICDEGs were predominantly localized to extracellular regions, extracellular space, cell periphery, plasma membrane, and vesicular structures (Fig. 5C, D). Regarding MF, ICDEGs were primarily associated with molecular function regulation, signaling receptor binding, molecular function activation, signaling receptor regulation, signaling receptor activation, and receptor ligand activity (Fig. 6A, B). Furthermore, ICDEGs exhibited significant enrichment in several crucial KEGG pathways, including cytokine-cytokine receptor interaction, cancer-related pathways, neuroactive ligand-receptor interaction, human cytomegalovirus infection, pathogenic Escherichia coli infection, natural killer cell-mediated cytotoxicity, TNF signaling pathway, AGE-RAGE signaling pathway in diabetic complications, and viral protein-cytokine/cytokine receptor interactions (Fig. 6C, D). Collectively, these findings suggest that ICDEGs’ functionality is intrinsically linked to immune cell regulation and activity.

### Identification and visualization of hub genes

**Fig. 7:**
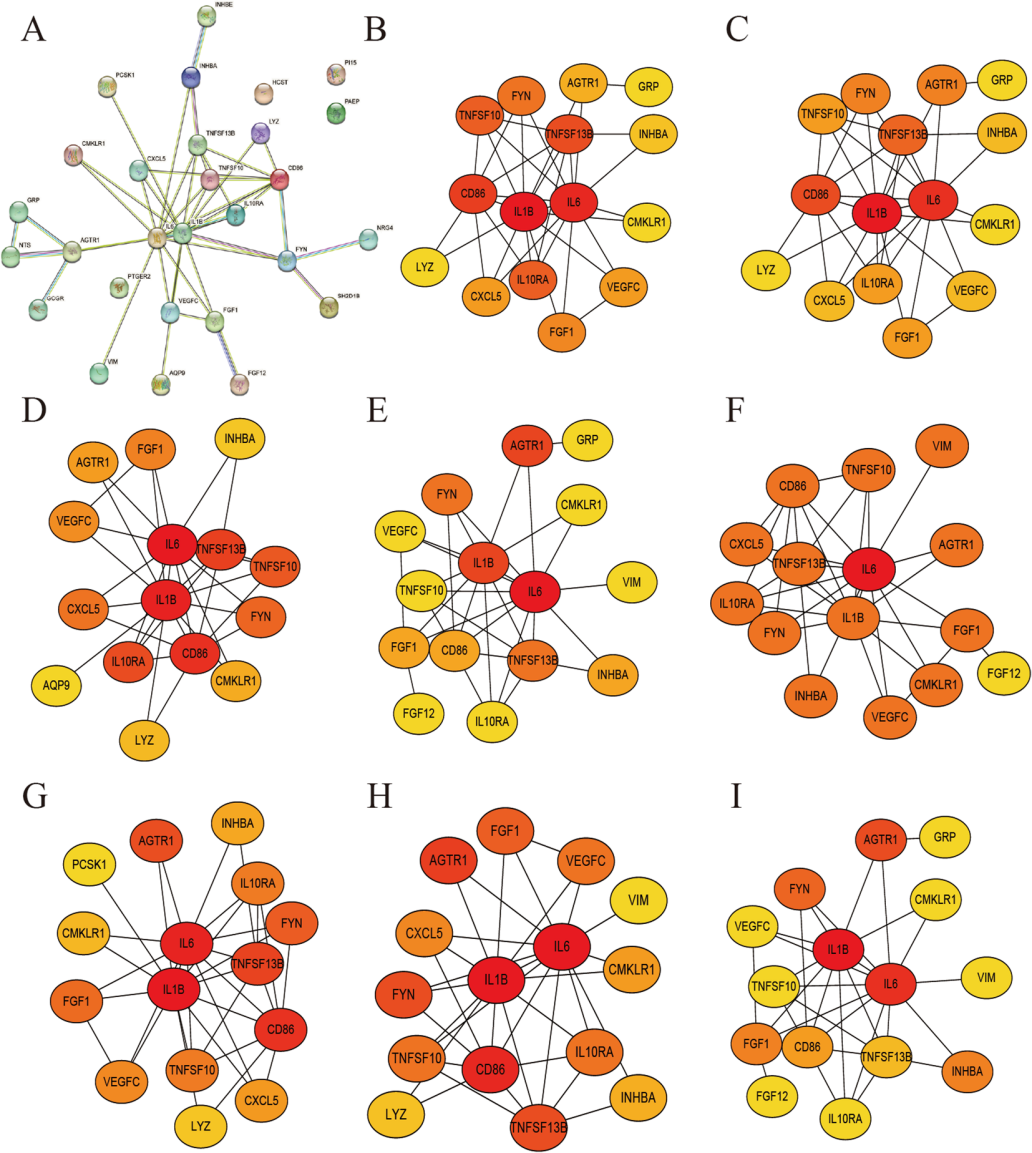
Identification of Hub genes through eight topological analysis algorithms implemented in the CytoHubba plugin. (**A**) PPI network of 28 ICDEGs generated using the STRING database; (**B**) MCC; (**C**) Degree; (**D**) EPC; (**E**) EcCentricity; (**F**) BottleNeck; (**G**) Closeness; (**H**) Radiality; (**I**) Betweenness. Protein ranking is represented by a color gradient (yellow to red), with darker red hues indicating higher-ranked proteins.

**Fig. 8:**
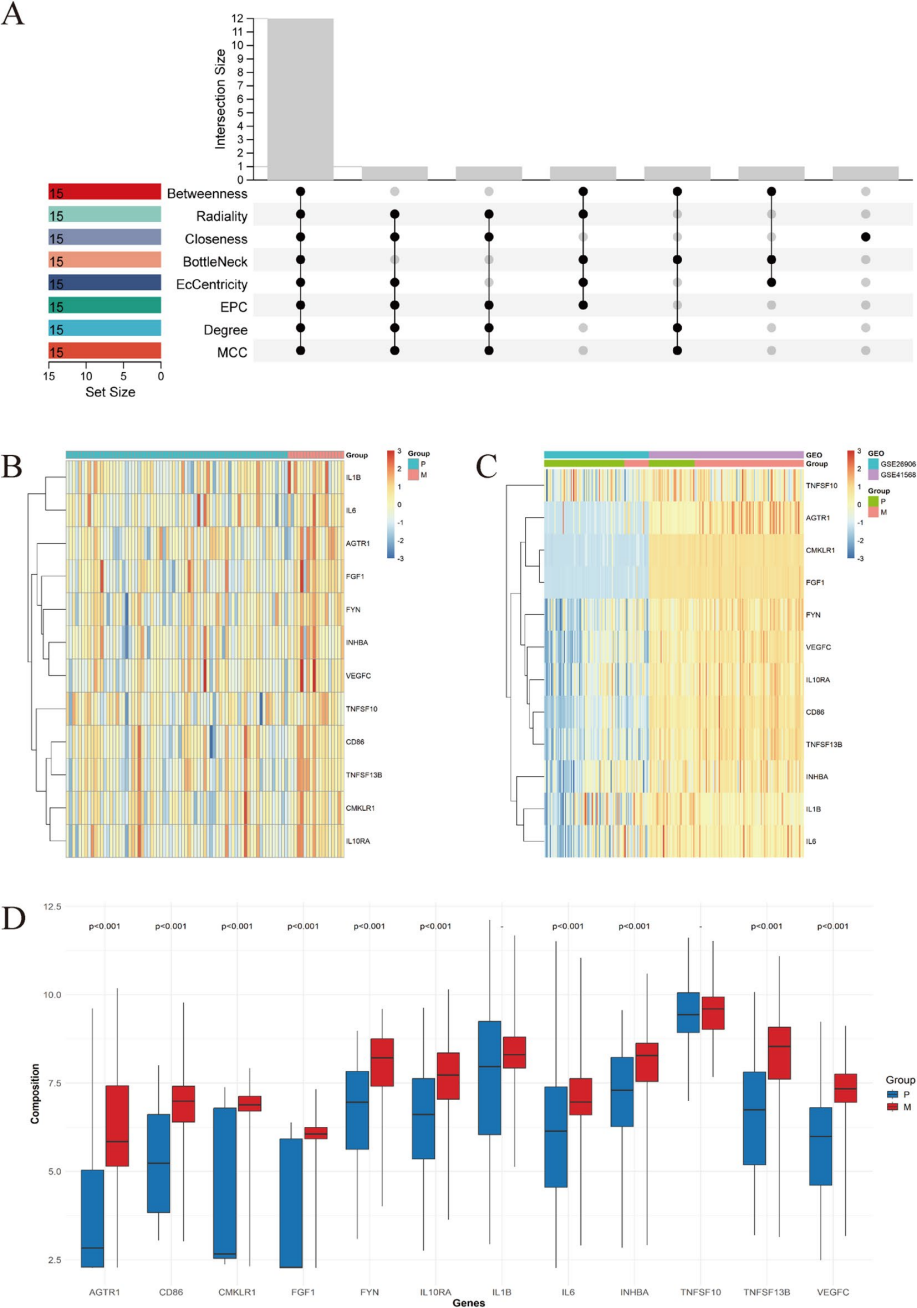
Identification and validation of Hub genes. (**A**) Hub gene identification using the R package “UpSet”; (**B**) Heatmap visualization of 12 Hub genes in the GSE33113 dataset; (**C**, **D**) Boxplot and heatmap representations of 12 Hub genes across the GSE26906 and GSE41568 datasets.

PPI networks of ICDEGs were constructed using the STRING database and subsequently visualized through Cytoscape software (Fig. 7A). Hub gene identification was performed by applying eight distinct topological analysis algorithms available in the CytoHubba plugin for Cytoscape, with the top 15 node genes being graphically represented (Fig. 7B-I). Implementation of the R package “UpSet” facilitated the identification of 12 Hub genes, all of which demonstrated upregulation in mCRC: *AGTR1*, *CD86*, *CMKLR1*, *FGF1*, *FYN*, *IL10RA*, *IL1B*, *IL6*, *INHBA*, *TNFSF10*, *TNFSF13B*, and *VEGFC*. Expression profiles of these genes within the GSE33113 microarray dataset were graphically represented as heatmaps (Fig. 8A, B). Validation of the 12 Hub gene expression patterns in the GSE26906 and GSE41568 datasets was conducted, with results visualized through box plots and heatmaps (Fig. 8C, D). The results showed that the expression of *AGTR1*, *CD86*, *CMKLR1*, *FGF1*, *FYN*, *IL10RA*, *IL6*, *INHBA*, *TNFSF13B*, and *VEGFC* was significantly higher in mCRC tissues than in CRC tissues. Therefore, it can be inferred that *AGTR1*, *CD86*, *CMKLR1*, *FGF1*, *FYN*, *IL10RA*, *IL6*, *INHBA*, *TNFSF13B*, and *VEGFC* can be used as candidate biomarkers for mCRC diagnosis.

### Characterization of the relationship between hub genes and mitochondrial function

**Fig. 9:**
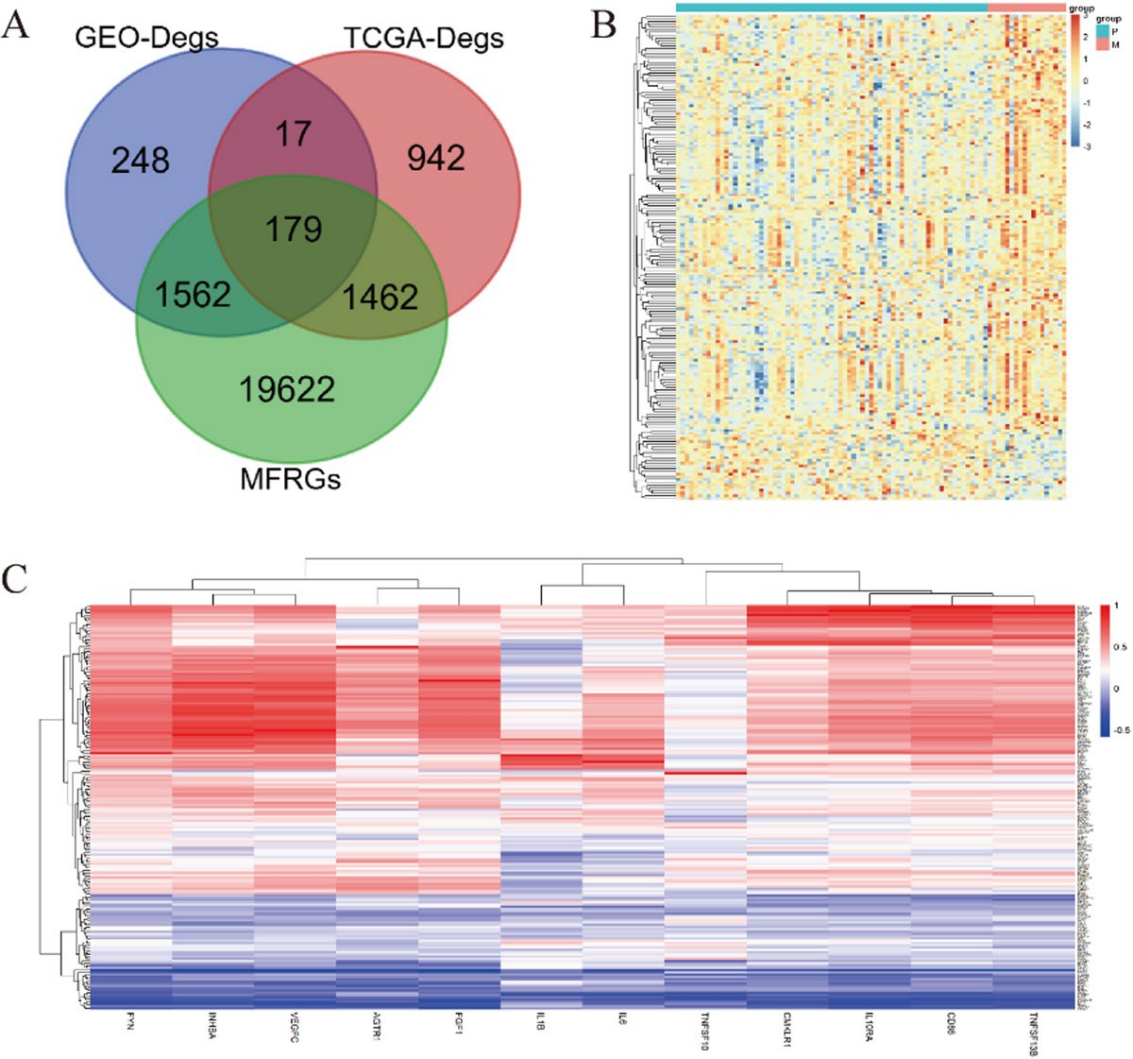
(**A**) Venn diagram illustrating the intersection of MFRGs with TCGA-DEGs and GEO-DEGs; (**B**) Heatmap visualization of 179 DE-MFRGs in the GSE33113 dataset; (**C**) Heatmap representation of Pearson correlation analysis between 12 Hub genes and DE-MFRGs.

Colorectal cancer cells undergo metabolic reprogramming to adapt to the tumor microenvironment, a process that includes modifications in mitochondrial functionality to fulfill the energetic and biosynthetic precursor requirements of malignant cells^50^. We investigated the relationship between Hub genes and MFRGs. From 22,825 MFRGs, 179 were identified as DE-MFRGs (Fig. 9A), comprising 25 upregulated and 154 downregulated genes. Pearson correlation analysis revealed significant associations (|*r*| ≥ 0.5, *p* < 0.05) between most DE-MFRGs and Hub genes (Fig. 9B, C), suggesting potential interplay between immune-related biomarkers and mitochondrial function in mCRC. This preliminary finding merits further investigation to elucidate its biological relevance.

### Assessment of the diagnostic validity of candidate biomarkers

**Fig. 10:**
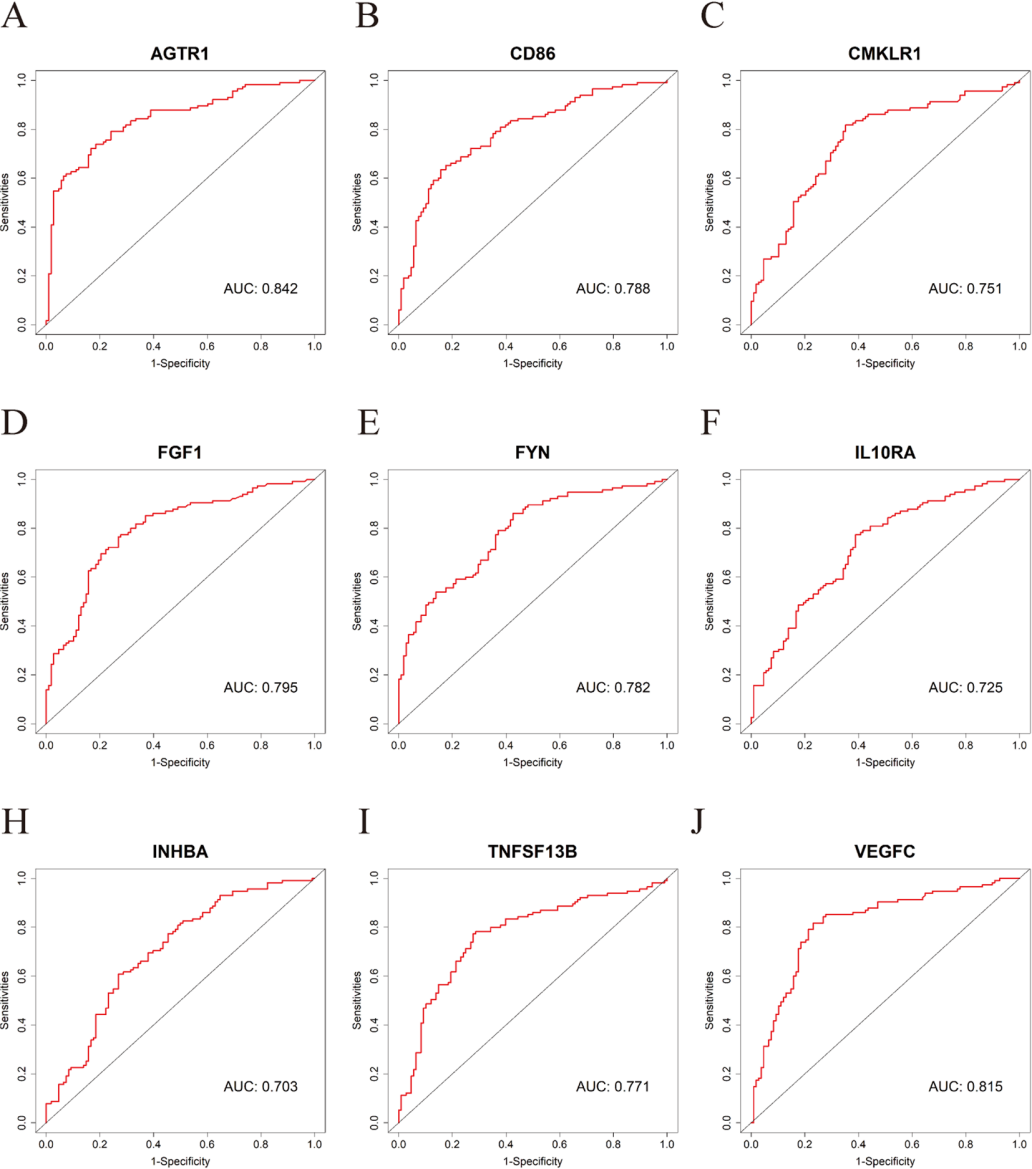
ROC curve analysis validating the diagnostic efficacy of 9 Hub genes for mCRC using GSE26906 and GSE41568 datasets. (**A**) AGTR1; (**B**) CD86; (**C**) CMKLR1; (**D**) FGF1; (**E**) FYN; (**F**) IL10RA; (**G**) INHBA; (**H**) TNFSF13B; (**I**) VEGFC.

Recent research has identified a distinct imbalance in antisense/sense RNA expression within the secretory intracellular FGF (iFGF) component in CRC. This study employed a comprehensive approach integrating transcriptome analysis of TCGA-COAD data, wet-lab validation, and AI-driven drug screening. Based on ROC curve analysis (AUC > 0.70), the iFGF component was established as a novel diagnostic biomarker^51^. To assess the diagnostic efficacy of the identified Hub genes for mCRC, we conducted ROC analysis using the GSE26906 and GSE41568 datasets. An AUC threshold > 0.700 was established as indicative of valid and highly reliable diagnostic performance^52^. The analysis revealed AUC values of 0.842, 0.788, 0.751, 0.795, 0.782, 0.725, 0.703, 0.771, and 0.815 for *AGTR1*, *CD86*, *CMKLR1*, *FGF1*, *FYN*, *IL10RA*, *INHBA*, *TNFSF13B*, and *VEGFC*, respectively (Fig. 10A-J). These findings demonstrate that these 9 Hub genes exhibit excellent sensitivity and specificity for mCRC diagnosis.

### Survival analysis of prognostic value for diagnostic biomarkers

**Fig. 11:**
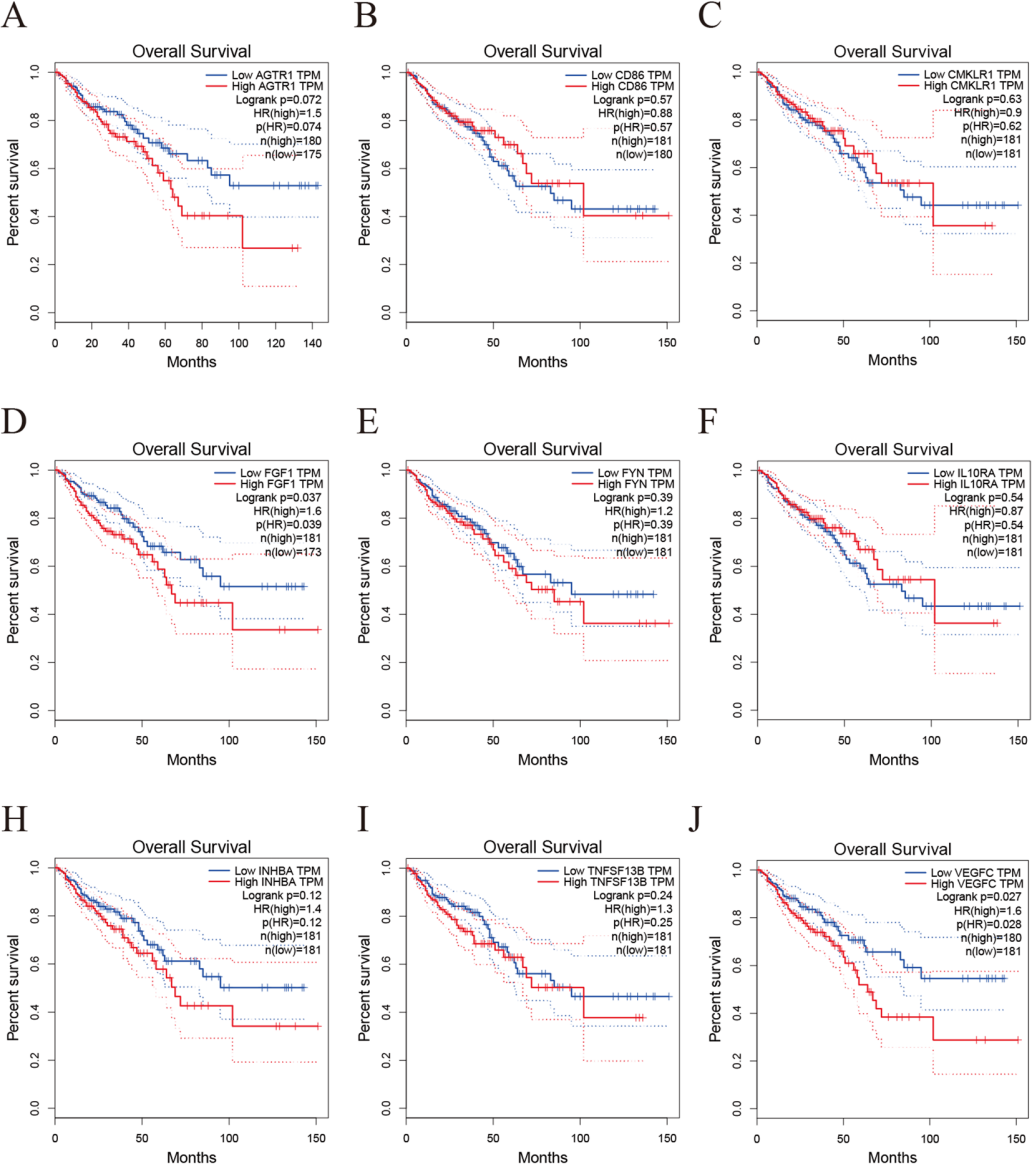
Kaplan-Meier survival analysis of the prognostic value of 9 Hub genes in the TCGA-CRC Dataset. (**A**) AGTR1; (**B**) CD86; (**C**) CMKLR1; (**D**) FGF1; (**E**) FYN; (**F**) IL10RA; (**G**) INHBA; (**H**) TNFSF13B; (**I**) VEGFC.

Kaplan-Meier survival analysis based on the TCGA-CRC cohort (Fig. 11A-J) revealed that among the 9 Hub genes, high expression of *FGF1* (*p* = 0.037, *HR* = 1.6) and *VEGFC* (*p* = 0.027, *HR* = 1.6) was significantly associated with poor prognosis in colorectal cancer patients (*p* < 0.05). Their negative impact on survival was primarily concentrated in the early-to-mid follow-up periods. Although *AGTR1* did not reach strict statistical significance (*p* = 0.072), it exhibited a clear trend towards high risk (*HR* = 1.5). Throughout the analysis period, the survival curve for the *AGTR1* high-expression group persistently lies below that of the low-expression group, suggesting its potential as a high-risk prognostic marker. Among the remaining genes, *INHBA* (*p* = 0.12, *HR* = 1.4) and *TNFSF13B* (*p* = 0.24, *HR* = 1.3) had Hazard Ratios greater than 1, indicating potential risk, but statistical power was limited due to lacked statistical significance of their survival curves. The survival curves for *CD86* (*HR* = 0.88), *CMKLR1* (*HR* = 0.9), *FYN* (*HR* = 1.2), and *IL10RA* (*HR* = 0.87) showed no significant separation (all *p* > 0.39).

### Relationship between diagnostic biomarkers and mCRC-infiltrating immune cells

**Fig. 12:**
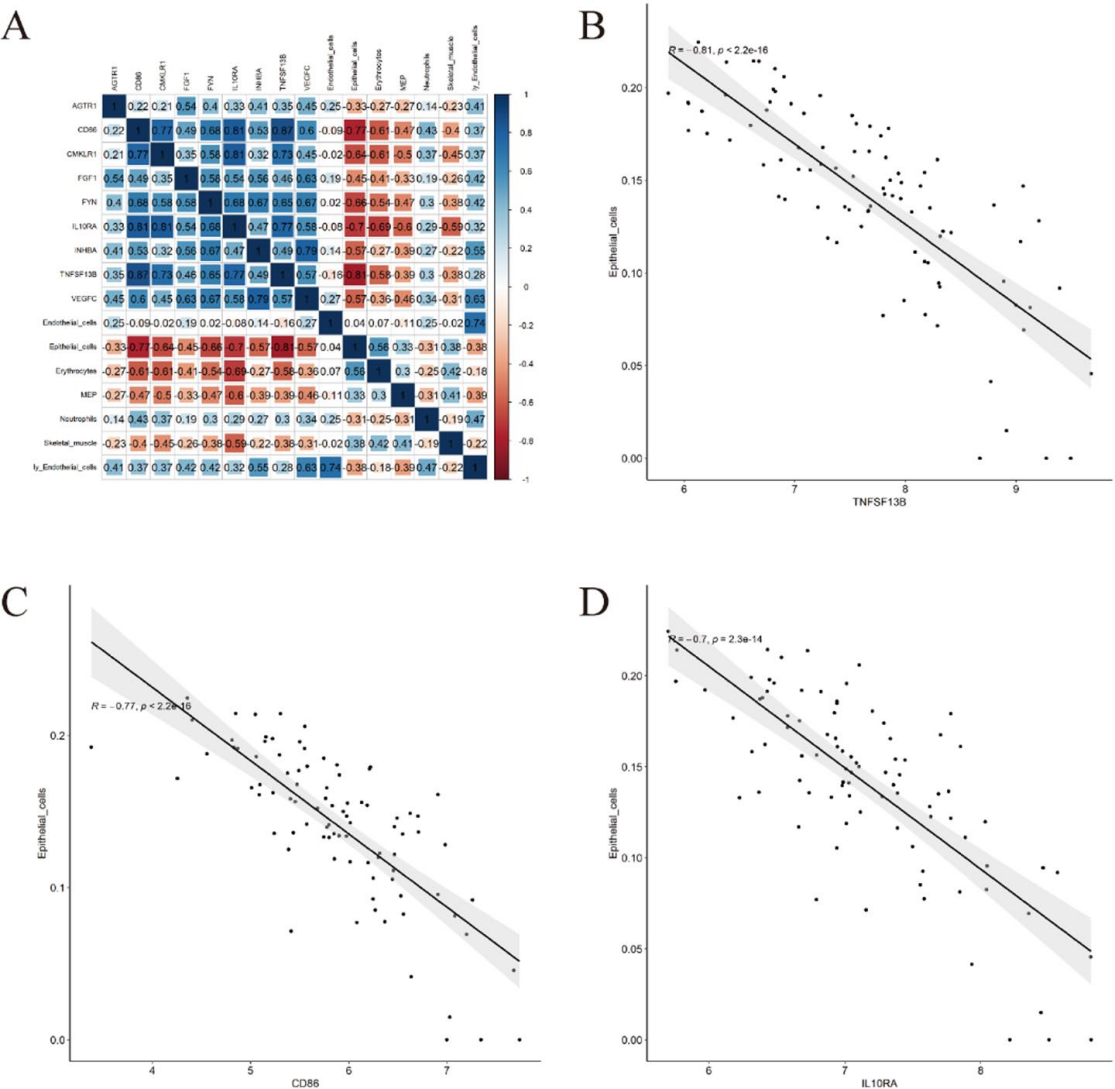
Associations between candidate diagnostic biomarkers and differentially infiltrating immune cell populations. (**A**) Correlation matrix illustrating relationships between candidate biomarkers and distinct infiltrating immune cell types; (**B**) Scatter plot demonstrating TNFSF13B-Epithelial cell correlation; (**C**) Scatter plot depicting CD86-Epithelial cell correlation; (**D**) Scatter plot showing IL10RA-Epithelial cell correlation.

We investigated the association between nine candidate diagnostic biomarkers (*AGTR1*, *CD86*, *CMKLR1*, *FGF1*, *FYN*, *IL10RA*, *INHBA*, *TNFSF13B*, and *VEGFC*) and seven distinct infiltrating immune cell populations (Endothelial cells, Epithelial cells, Erythrocytes, MEP, Neutrophils, Skeletal muscle cells, and Lymphatic endothelial cells). Applying a threshold of |*r*| > 0.70 with *p* < 0.05, significant correlations were identified (Fig. 12A). Scatter plot analysis revealed strong negative correlations between Epithelial cells and *TNFSF13B* (*r* = -0.81), *CD86* (*r* = -0.77), and *IL10RA* (*r* = -0.70) (Fig. 12B-D).

## Discussion

mCRC represents a highly aggressive malignancy whose development is intricately associated with multiple factors, including genetic predisposition, environmental determinants, and alterations in the TME^53^. The progression of mCRC is frequently characterized by complex immune evasion mechanisms that enable tumor cells to circumvent host immune surveillance, thereby facilitating tumor proliferation and metastatic dissemination^54^. Emerging evidence indicates that the activation and interplay of infiltrating immune cells are pivotal in tumor progression and metastatic processes, suggesting that therapeutic strategies targeting the immune microenvironment may represent a novel approach for mCRC management^55^. Nevertheless, significant challenges persist in the diagnosis and therapeutic management of mCRC, particularly in the identification of reliable diagnostic biomarkers^56^. Conventional detection methods frequently lack adequate sensitivity and specificity, necessitating the development of novel biomarkers to enhance early diagnostic accuracy. High-throughput sequencing technologies offer innovative approaches for investigating tumorigenesis and progression^57^enabling comprehensive exploration of gene expression alterations and their underlying mechanisms in mCRC. This investigation employed an integrative approach combining high-throughput sequencing and bioinformatics methodologies to elucidate the role of infiltrating immune cells in mCRC pathogenesis, with the dual objectives of identifying novel diagnostic biomarkers and establishing a theoretical framework for early detection and personalized therapeutic strategies in mCRC.

### Collective significance of altered immune and stromal populations

The integrated immune deconvolution using both ssGSEA and xCell revealed a profoundly remodeled TME in mCRC. The enrichment of endothelial cells and lymphatic endothelial cells populations underscores the critical role of neovascularization in supporting metastatic growth and dissemination. The significant increase in neutrophils points towards a dominant pro-tumorigenic, immunosuppressive inflammation within the mCRC TME^58^. Conversely, the reduction in epithelial cells signifies widespread EMT and loss of tissue architecture, fundamental to the invasive and metastatic phenotype^59^. The decrease in erythrocytes and MEP highlights the frequent occurrence of tumor-associated anemia and potential bone marrow suppression in advanced disease^60,61^while skeletal muscle loss may reflect cancer cachexia^62^. These collective alterations in specific immune and stromal compartments, driven in part by the dysregulated Hub genes identified herein, create a TME permissive for metastasis and resistant to immune attack.

### Mechanistic roles of hub genes in metastasis and TME remodeling

To identify more robust diagnostic biomarkers, we systematically screened 2599 and 2005 differentially expressed genes (DEGs), including 28 immune-related DEGs (ICDEGs), from CRC and mCRC samples in the TCGA and GEO databases, respectively. Functional enrichment analyses through GO and KEGG pathways revealed that these ICDEGs were predominantly associated with immune responses, suggesting their potential involvement in immune cell regulation. Subsequent validation of 9 Hub genes (*AGTR1*, *CD86*, *CMKLR1*, *FGF1*, *FYN*, *IL10RA*, *INHBA*, *TNFSF13B*, and *VEGFC*) using independent test sets and ROC regression analyses demonstrated their excellent diagnostic performance, thereby reinforcing the reliability of our findings. Beyond their diagnostic significance identified in this study, our 9 Hub genes likely function as central orchestrators of metastatic progression and TME remodeling through specific molecular mechanisms.

#### Angiogenesis and lymphangiogenesis

*VEGFC* (Vascular Endothelial Growth Factor C) is a crucial cytokine belonging to the Vascular Endothelial Growth Factor (VEGF) family. *VEGFC* is the key factor regulating lymphangiogenesis by binding to VEGFR-3 on lymphatic endothelial cells (LECs)^63^. This directly explains the significant enrichment of LECs we observed in mCRC tissues (Fig. 4G). The newly formed lymphatic vessels provide pathways for tumor cells to spread to lymph nodes and distant sites, which is a critical step in metastasis^64^. Furthermore, the VEGFC/VEGFR3 axis modulates tumor-associated macrophages (TAMs), and they synergistically suppress anti-tumor immunity and promote immune escape^65^.

*FGF1* (Fibroblast Growth Factor 1) is a potent pro-angiogenic factor that stimulates endothelial cell proliferation, migration, and the formation of new blood vessels^66^. This angiogenic switch supports the metabolic demands of both primary and metastatic lesion growth. Our finding of elevated endothelial cells in mCRC (Fig. 4E) is consistent with the role of *FGF1*. *FGF1* also directly promotes CRC cell proliferation and migration via the mTOR-S6K1 signaling pathway^67^linking angiogenesis to the intrinsic metastatic potential of tumor cells. Studies demonstrate that *AKR1B10* inhibits *FGF1* and, in an FGF1-dependent manner, suppresses the proliferative and migratory capacities of CRC cells. *AKR1B10* exerts a tumor-suppressive effect in CRC by inactivating *FGF1* and represents a novel target for combination therapy in CRC^68^.

#### Epithelial–mesenchymal transition (EMT) and invasion

*INHBA* (Inhibin Beta A), a member of the inhibin family, promotes EMT, invasion, and metastasis through the SMAD-dependent signaling pathway. High *INHBA* expression is closely associated with poor prognosis in CRC patients. In vitro knockdown of *INHBA* suppresses the proliferation, migration, and invasion of CRC cells. Mechanistically, high INHBA expression has also been found to activate the TGF-β pathway^69^. Both the INHBA gene and the lncRNA *PELATON* have emerged as critical factors in CRC progression. Exploring the immunological roles of *INHBA* and *PELATON* in CRC was achieved by combining computational prediction with experimental validation^70^.

*FYN* (FYN proto-oncogene, Src family tyrosine kinase) is a Src family kinase. In T cells, *FYN* participates in T cell receptor (TCR)-mediated signal transduction and is a key molecule for T cell activation^71^. Additionally, functional in vitro experiments demonstrate that *IBSP* promotes the growth and aggressiveness of CRC, and the underlying mechanism involves *IBSP* enhancing the aberrant activation of the oncogenic CRC FYN/β-catenin signaling pathway^72^. This directly increases tumor cell invasive capacity, which is crucial for metastasis. Furthermore, PrPc has been shown to regulate *SATB1* expression via the Fyn-SP1 pathway. Given that *SATB1* is recognized as a key protein controlling tumor development and progression, knockdown of PrPc reduces the metastatic capacity of colorectal cancer cells and decreases distant metastasis in vivo^73^.

*AGTR1* (Angiotensin II Receptor Type 1) is a primary receptor for angiotensin II and belongs to the G protein-coupled receptor (GPCR) family. The binding of angiotensin II to *AGTR1* activates multiple signaling pathways, promoting cancer cell survival and proliferation^74^. Studies demonstrate that *AGTR1* promotes tumor cell migration and invasion, facilitating metastasis by influencing extracellular matrix remodeling and cell-cell interactions^75^.

#### Immune suppression and evasion

*CD86* (Cluster of Differentiation 86), also known as B7-2, is a critical co-stimulatory molecule. Within the tumor microenvironment (TME), *CD86* binds to *CD28* and *CTLA-4* on the surface of T cells, regulating T cell activation, proliferation, and survival^76^. *CD86* expression is found on tumor-associated macrophages and dendritic cells; by modulating the function of these cells, *CD86* influences the composition of the tumor microenvironment, thereby potentially promoting or suppressing tumor development^77^. Its significant negative correlation with epithelial cell abundance (Fig. 12C) may reflect its association with immune cell activity within the TME or altered epithelial-immune interplay.

*IL10RA* (Interleukin-10 Receptor Alpha), a subunit of the interleukin-10 (IL-10) receptor, primarily mediates the immunomodulatory effects of IL-10. Following binding with IL-10, *IL10RA* activates the downstream JAK-STAT signaling pathway-particularly STAT3-which is critical for regulating immune responses and suppressing inflammation^78^. Through the JAK-STAT pathway (specifically STAT3), IL-10 signaling inhibits dendritic cell maturation, reduces pro-inflammatory cytokine production by macrophages and other cells, and attenuates T-cell activation, thereby effectively suppressing anti-tumor immunity. Its negative correlation with epithelial cells (Fig. 12D) may be related to IL-10’s role in inhibiting epithelial inflammation or modulating epithelial barrier function.

*TNFSF13B* (Tumor Necrosis Factor Ligand Superfamily Member 13B), commonly known as *APRIL* (A Proliferation-Inducing Ligand), is a cytokine belonging to the tumor necrosis factor (TNF) superfamily. *TNFSF13B* promotes B-cell survival and proliferation, particularly during infection and immune responses. As a key cytokine, *TNFSF13B* participates in regulating adaptive immune responses, promoting antibody production and the formation of immune memory^79^. In solid tumors, *APRIL* signaling can foster a pro-tumor humoral milieu and may modulate myeloid cell function, leading to immune suppression. Its strong negative correlation with epithelial cells (Fig. 12B) suggests its potential involvement in processes affecting epithelial integrity or EMT, with evidence supporting reduced epithelial phenotype upon diminished *BAFF/APRIL* signaling^80^.

*CMKLR1* (Chemokine-like receptor 1), also known as chemokine receptor-like 1, is a chemokine receptor. *CMKLR1* primarily promotes the migration of immune cells by binding to chemerin^81^. Depending on the microenvironment, it can exert either pro-inflammatory or anti-inflammatory effects, influencing TME composition. *CMKLR1* may play a multifunctional role in CRC pathogenesis, potentially through its effects on tumor budding and peritumoral lymphocyte infiltration^82^. Furthermore, studies indicate that *CMKLR1* activity may be linked to CRC angiogenesis via *MMP-9* activity^83^.

### Synergistic effects within the TME

Dysregulation of these hub genes likely creates a synergistic, self-reinforcing network driving mCRC progression. *VEGFC*, *FGF1*, *INHBA*, *FYN*, and *AGTR1* promote angiogenesis, lymphangiogenesis, EMT, invasion, and tumor cell survival – constituting pro-metastatic signaling. *CD86*, *IL10RA*, *TNFSF13B*, and *CMKLR*1 suppress effective anti-tumor immunity while simultaneously potentially promoting pro-tumor immune subsets – representing immunosuppressive signaling. Collectively, these signals explain the observed TME alterations: enrichment of pro-metastatic and pro-angiogenic cells (Fig. 4E-G) and reduction of epithelial cells (Fig. 4A); the negative correlations of key hub genes *TNFSF13B*, *CD86*, and *IL10RA* with epithelial cells (Fig. 12B-D) provide direct evidence linking these genes to TME remodeling; the enrichment of neutrophils (Fig. 4F) may be driven by cytokines downstream of hub genes and contributes to inflammation, stromal remodeling, and immune suppression^58^.

### Implications and future directions

Therapeutic strategies targeting these hub genes or their downstream effectors hold significant promise. For instance, angiotensin receptor blockers (ARBs) targeting *AGTR1*^84^, anti-angiogenic therapies targeting *VEGFC/FGF1*, or immune checkpoint modulators targeting *CD86*^85^ could be explored in mCRC. The significant association of high *FGF1* and *VEGFC* expression with poor prognosis (Fig. 11D, J) further underscores their clinical relevance. However, further in vitro and in vivo studies are essential to fully elucidate the precise molecular mechanisms and validate these hub genes as therapeutic targets. In conclusion, we propose that these nine diagnostic biomarkers may contribute to mCRC pathogenesis through interactions with relevant immune cell populations. Additionally, the observed correlations between Hub genes and mitochondrial function-related genes (Fig. 9C) imply a potential link between immune dysregulation and metabolic adaptation in mCRC, though the exact biological significance requires validation.

## Conclusion

### Strengths and limitations

This study possesses several notable strengths. Firstly, it represents the first application of the xCell algorithm to comparatively analyze immune infiltration patterns specifically between primary CRC and mCRC, providing novel insights into the evolving tumor immune microenvironment during metastasis. Secondly, we employed a robust, multi-dimensional bioinformatics framework integrating data from multiple independent datasets (TCGA-CRC, GSE33113, GSE26906, GSE41568) and utilizing diverse analytical approaches (differential expression, immune cell deconvolution with ssGSEA and xCell, functional enrichment, PPI network analysis, hub gene identification, ROC validation, survival analysis, correlation studies). This comprehensive strategy enhances the reliability of our findings. Thirdly, we successfully identified and validated 9 novel immune-related Hub genes (*AGTR1*, *CD86*, *CMKLR1*, *FGF1*, *FYN*, *IL10RA*, *INHBA*, *TNFSF13B*, and *VEGFC*) as promising diagnostic biomarkers for mCRC, several of which (*AGTR1*, *CD86*, *CMKLR1*, *TNFSF13B*) have been rarely reported in this context. Finally, our exploration of the correlations between these biomarkers and specific infiltrating immune cell populations, particularly the significant inverse relationships between epithelial cells and *TNFSF13B*, *CD86*, and *IL10RA*, offers mechanistic hypotheses for their involvement in mCRC pathogenesis within the tumor microenvironment.

However, several limitations warrant consideration. Primarily, the retrospective nature of this investigation, relying on publicly available datasets, necessitates validation in prospective clinical cohorts with larger sample sizes and more detailed clinical annotations to substantiate the diagnostic and prognostic utility of the identified biomarkers. Secondly, while we identified key genes and correlations, the upstream and downstream regulatory mechanisms governing the expression and function of these nine hub genes, as well as their precise interactions with the infiltrating immune cells, require further comprehensive molecular and cellular exploration. Lastly, extensive in vivo and in vitro experimentation, encompassing human sample analyses and animal model studies, should be conducted prior to clinical translation of these findings.

Despite these limitations, this investigation provides comprehensive insights into the distinct immune infiltration landscape of mCRC and identifies 9 potential diagnostic biomarkers. The significant associations established between these biomarkers, suggesting their interplay may play a pivotal role in mCRC progression by modulating immune responses. Collectively, this study offers novel perspectives on the immunoregulatory mechanisms underlying mCRC and establishes new avenues for future diagnostic and therapeutic research in this field.

## Supplementary Information

Below is the link to the electronic supplementary material.

Supplementary Material 1

Supplementary Material 2

## Data Availability

The TCGA-CRC, GSE33113, GSE26906 and GSE41568 datasets were publicly available and obtained from The Cancer Genome Atlas Program (TCGA, https://portal.gdc.cancer.gov/) and Gene Expression Omnibus (GEO, https://www.ncbi.nlm.nih.gov/geo/). The names of the repository and accession number(s) can be found in the online repositories.
