# Supplementary figures and images for: Exploration of effective biomarkers and infiltrating immune cells in metastatic colorectal cancer based on bioinformatics analysis
Source: Sci Rep. 2025 Sep 26;15:33156. doi: 10.1038/s41598-025-18589-4 (PMC12474896; doi:10.1038/s41598-025-18589-4)

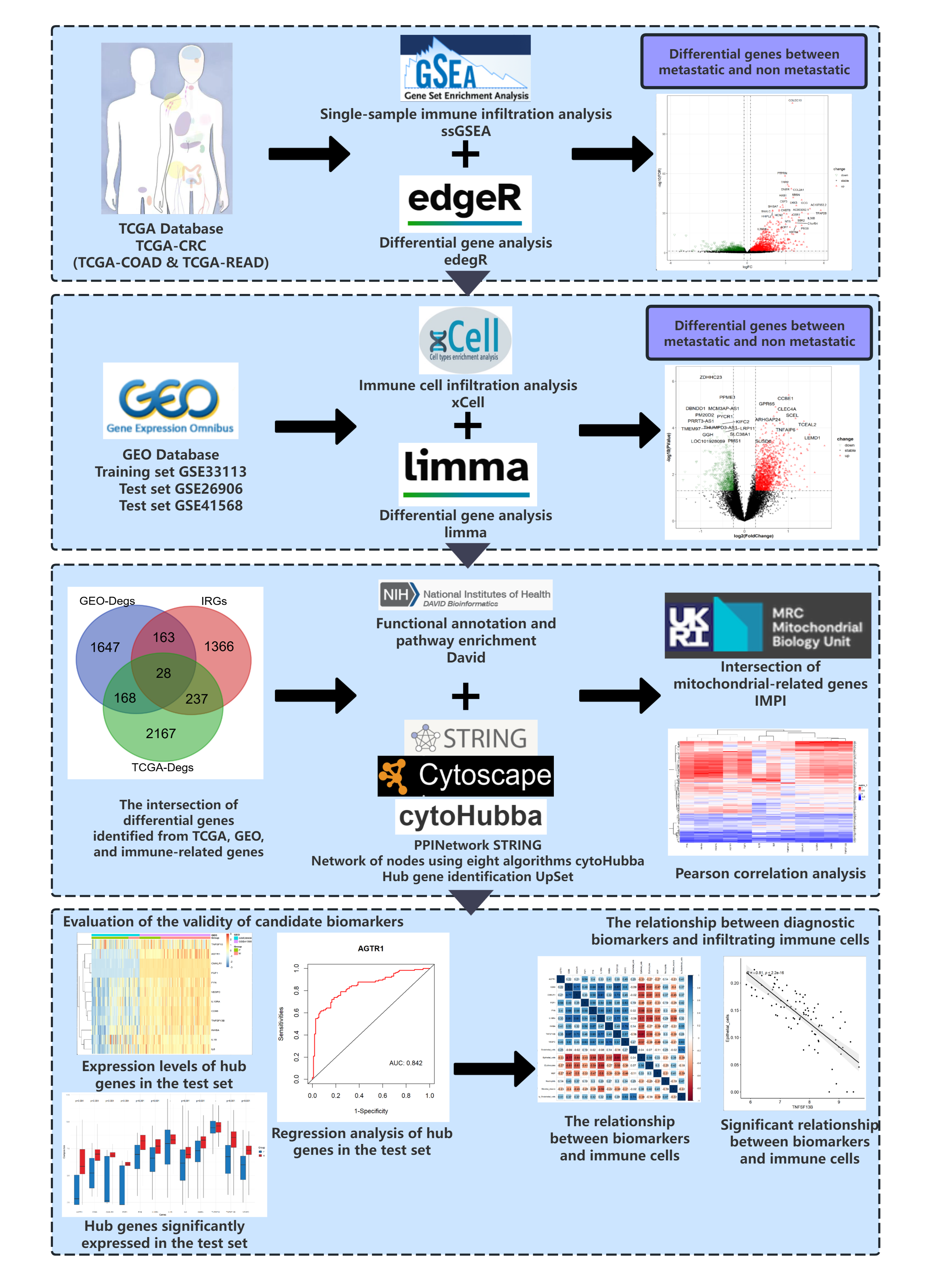

Supplement: Supplementary file 1 — Supplementary Material 1 [file 41598_2025_18589_MOESM1_ESM.zip › Figure 1.png]

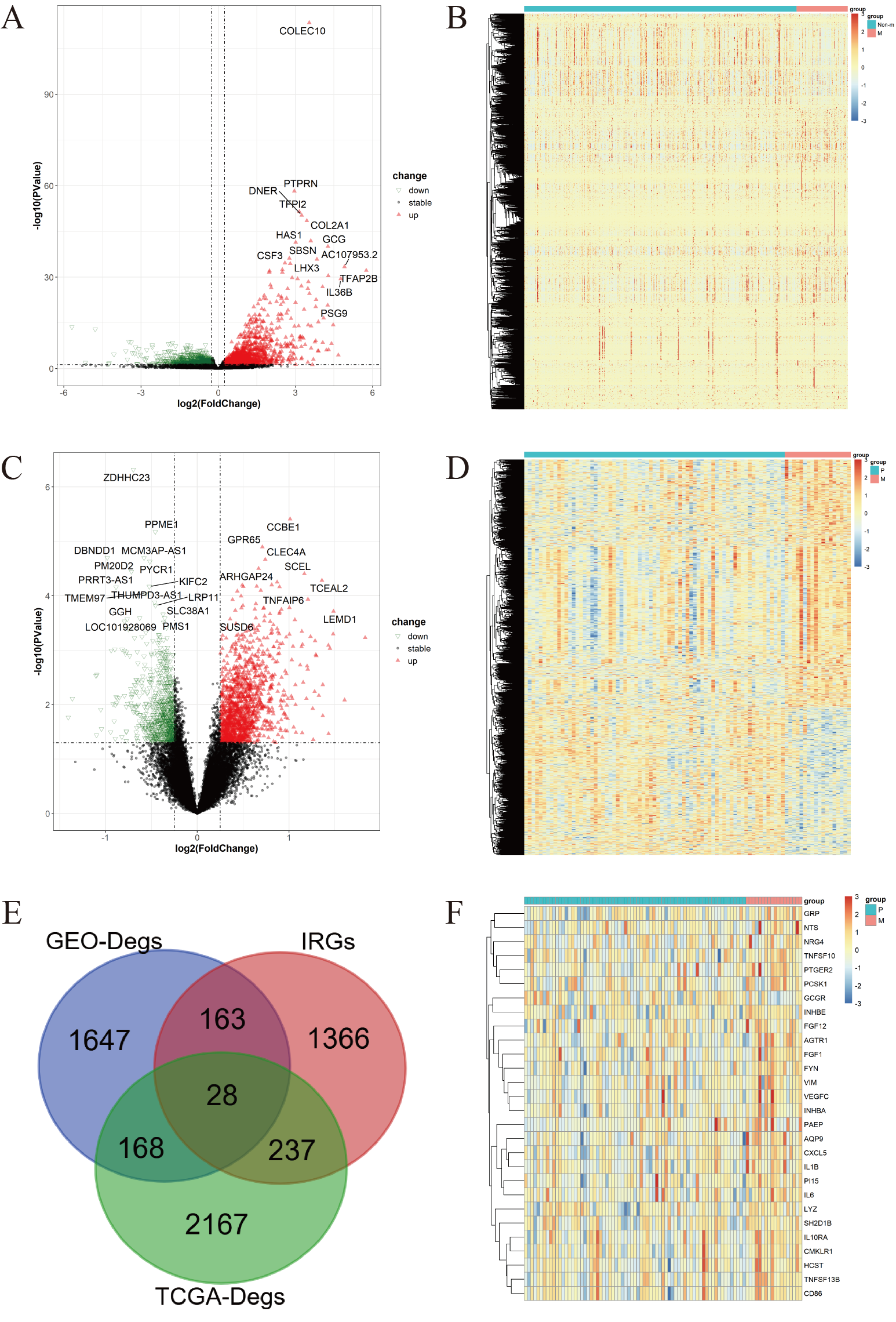

Supplement: Supplementary file 1 — Supplementary Material 1 [file 41598_2025_18589_MOESM1_ESM.zip › Figure 2.bmp]

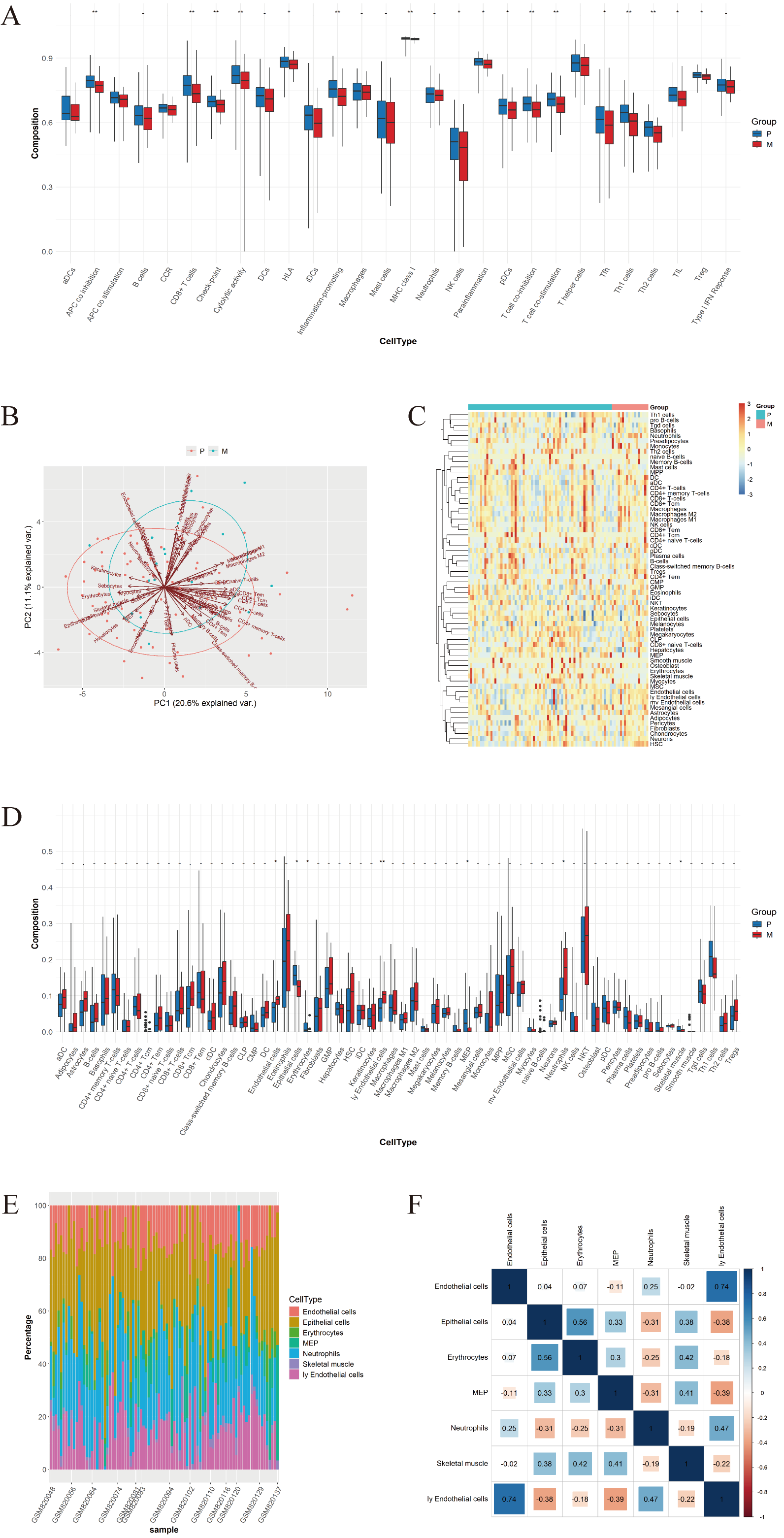

Supplement: Supplementary file 1 — Supplementary Material 1 [file 41598_2025_18589_MOESM1_ESM.zip › Figure 3.bmp]

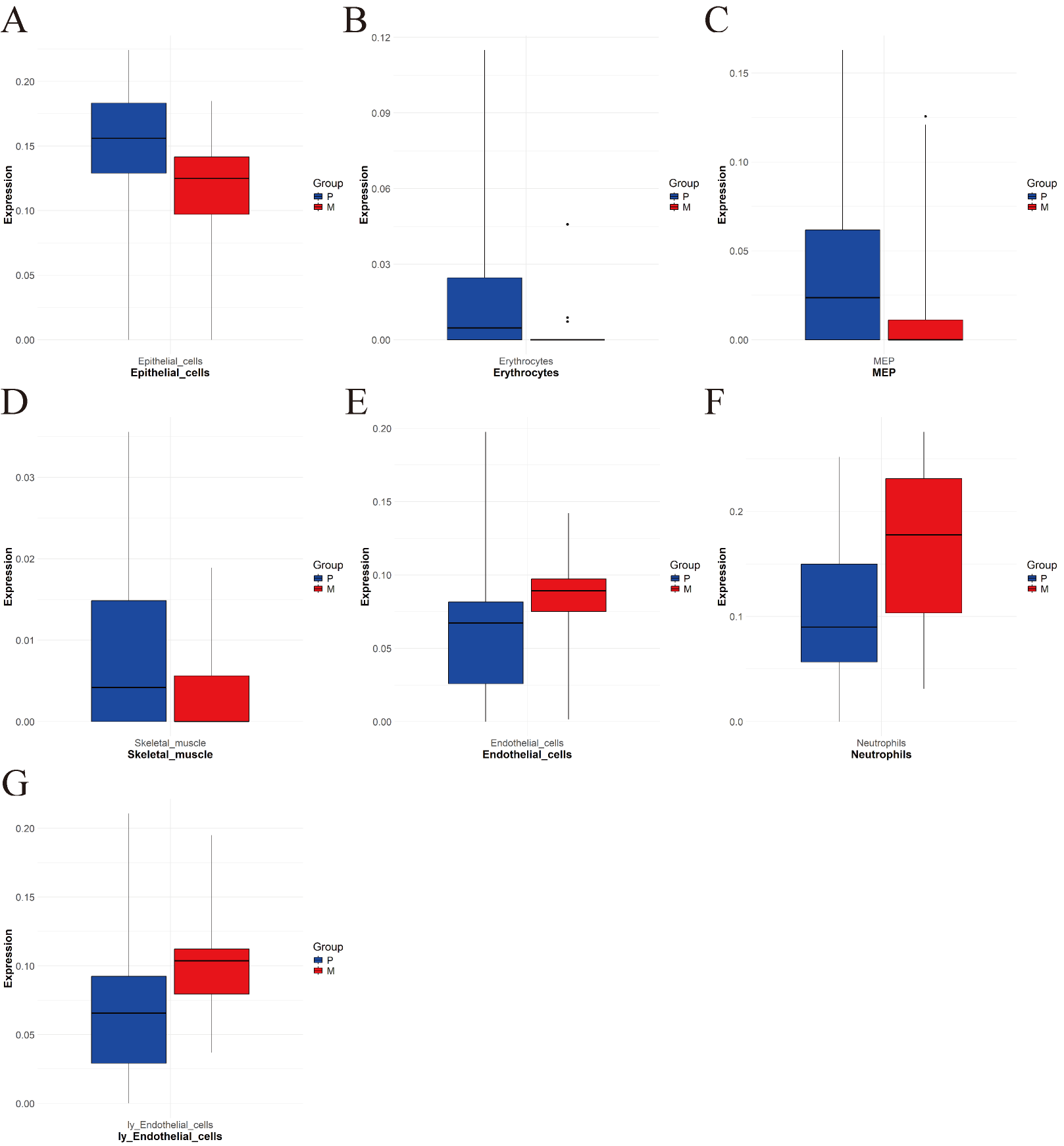

Supplement: Supplementary file 1 — Supplementary Material 1 [file 41598_2025_18589_MOESM1_ESM.zip › Figure 4.bmp]

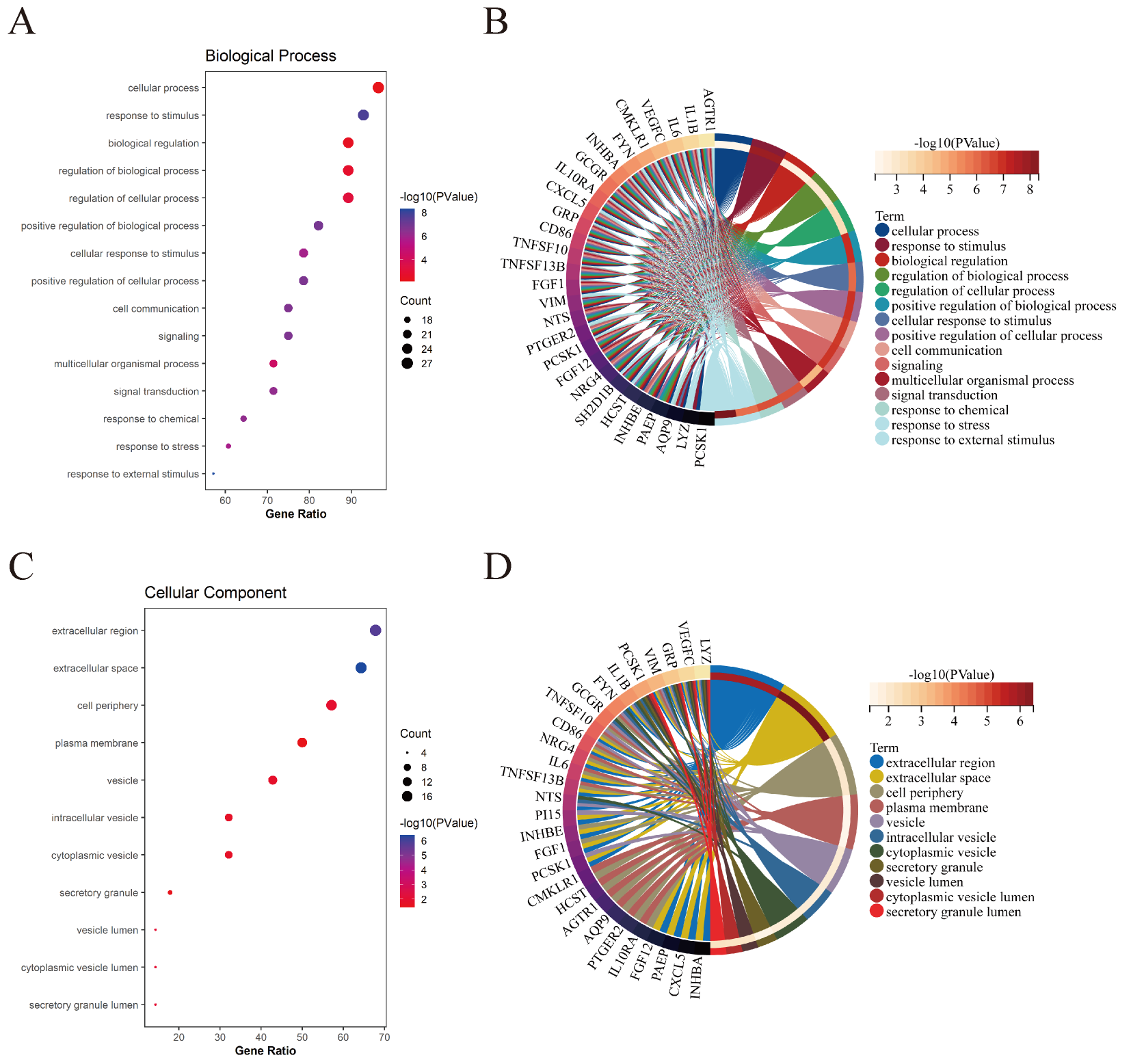

Supplement: Supplementary file 1 — Supplementary Material 1 [file 41598_2025_18589_MOESM1_ESM.zip › Figure 5.bmp]

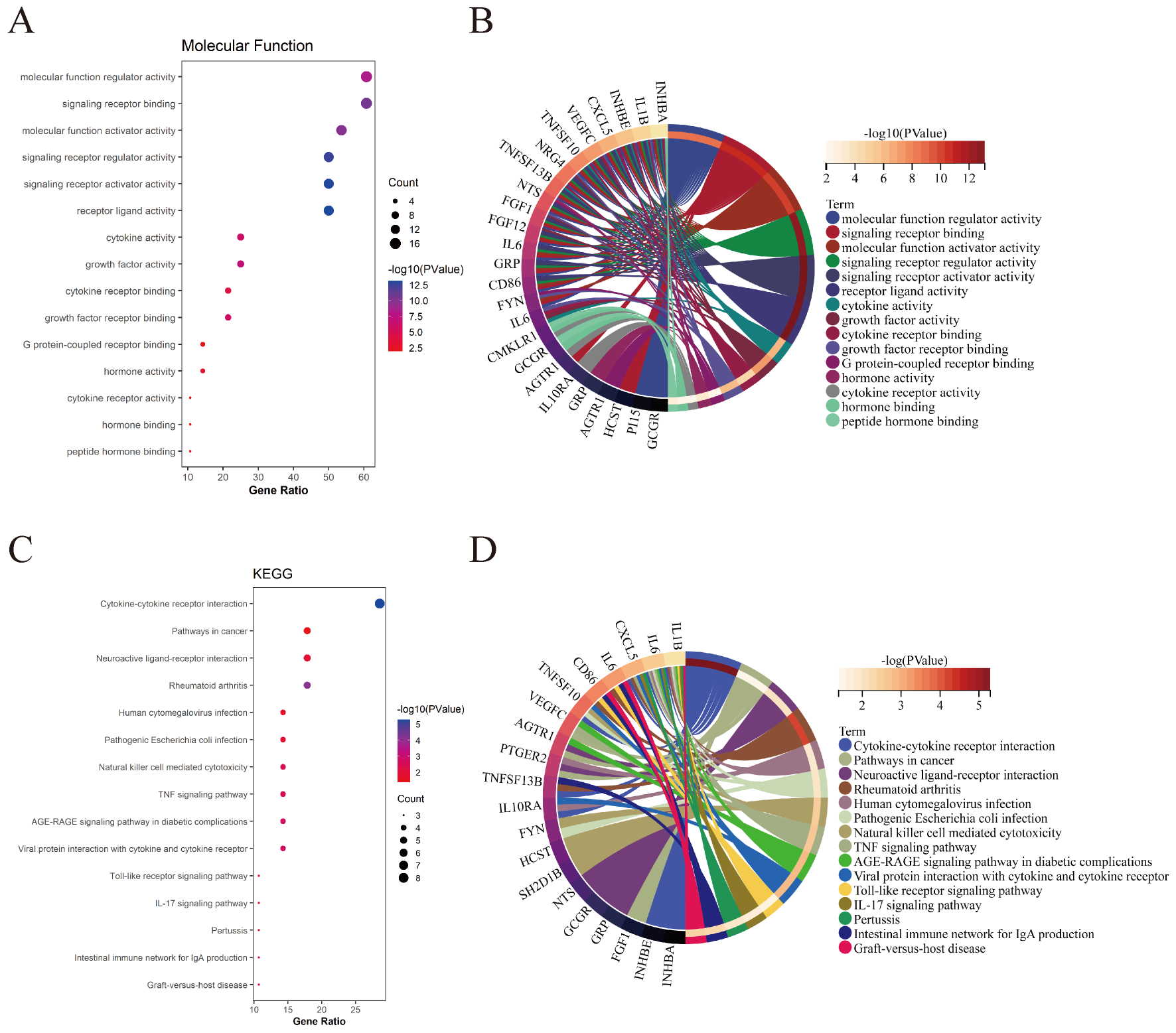

Supplement: Supplementary file 1 — Supplementary Material 1 [file 41598_2025_18589_MOESM1_ESM.zip › Figure 6.bmp]

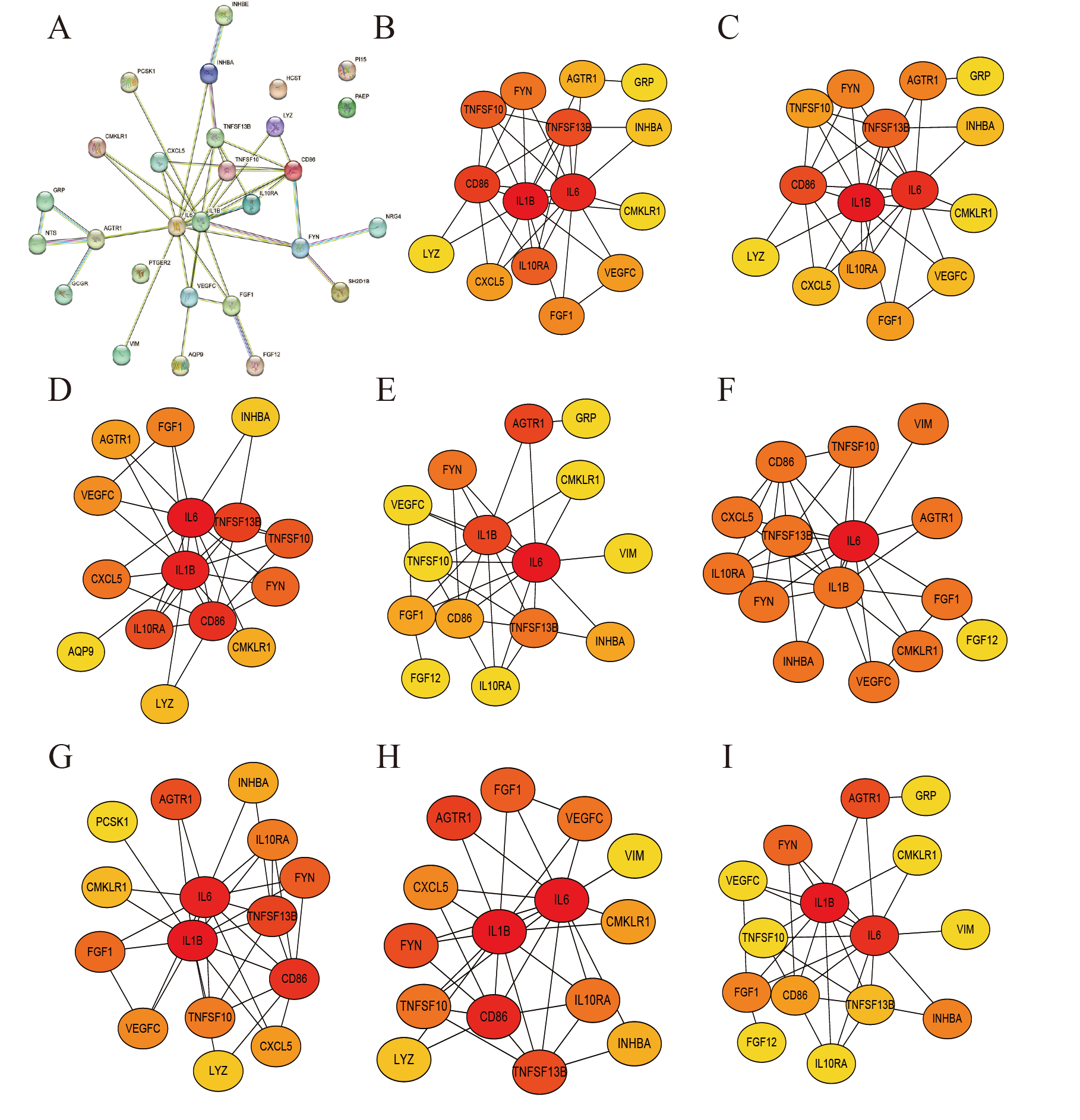

Supplement: Supplementary file 1 — Supplementary Material 1 [file 41598_2025_18589_MOESM1_ESM.zip › Figure 7.bmp]

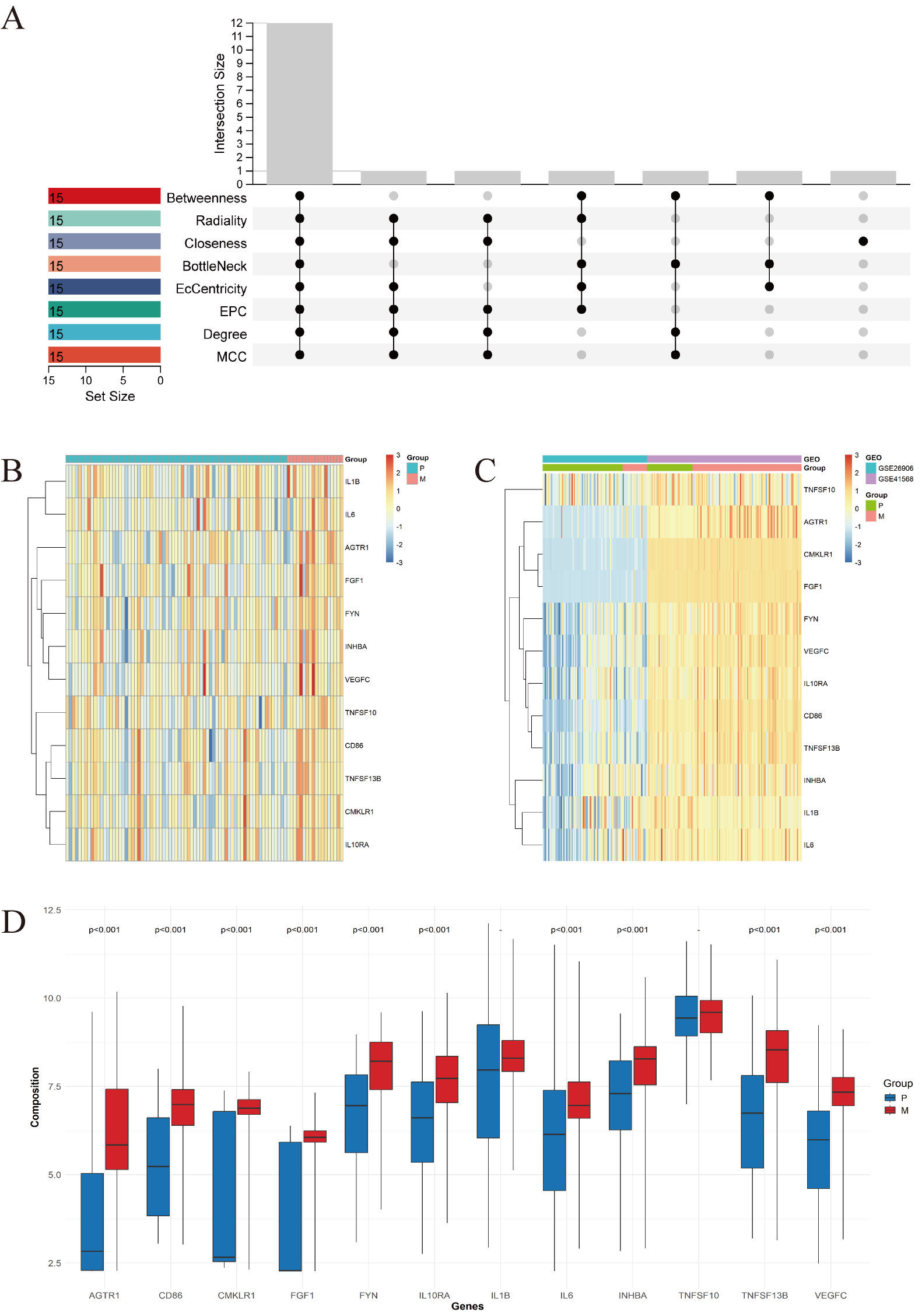

Supplement: Supplementary file 1 — Supplementary Material 1 [file 41598_2025_18589_MOESM1_ESM.zip › Figure 8.bmp]

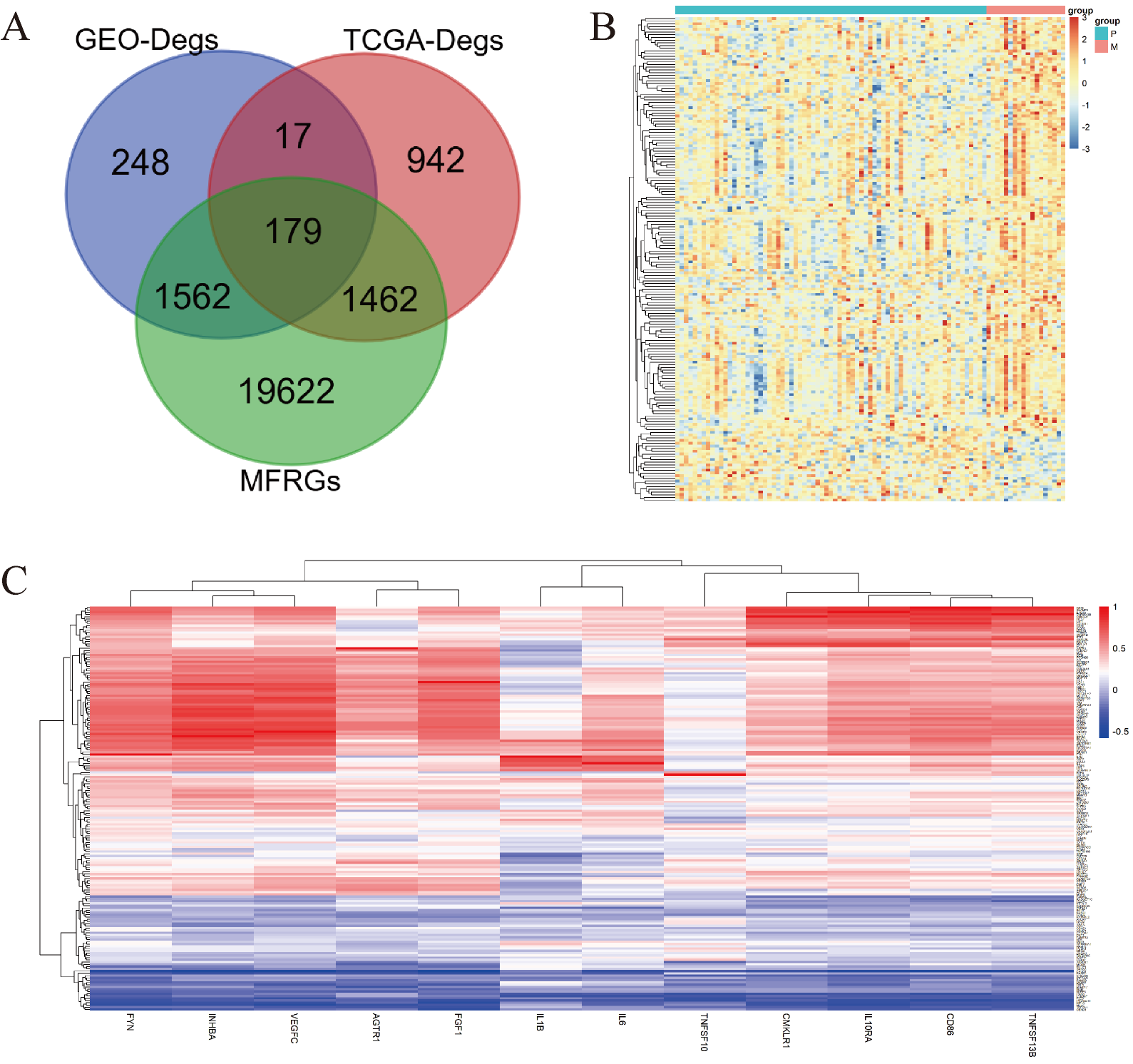

Supplement: Supplementary file 1 — Supplementary Material 1 [file 41598_2025_18589_MOESM1_ESM.zip › Figure 9.bmp]

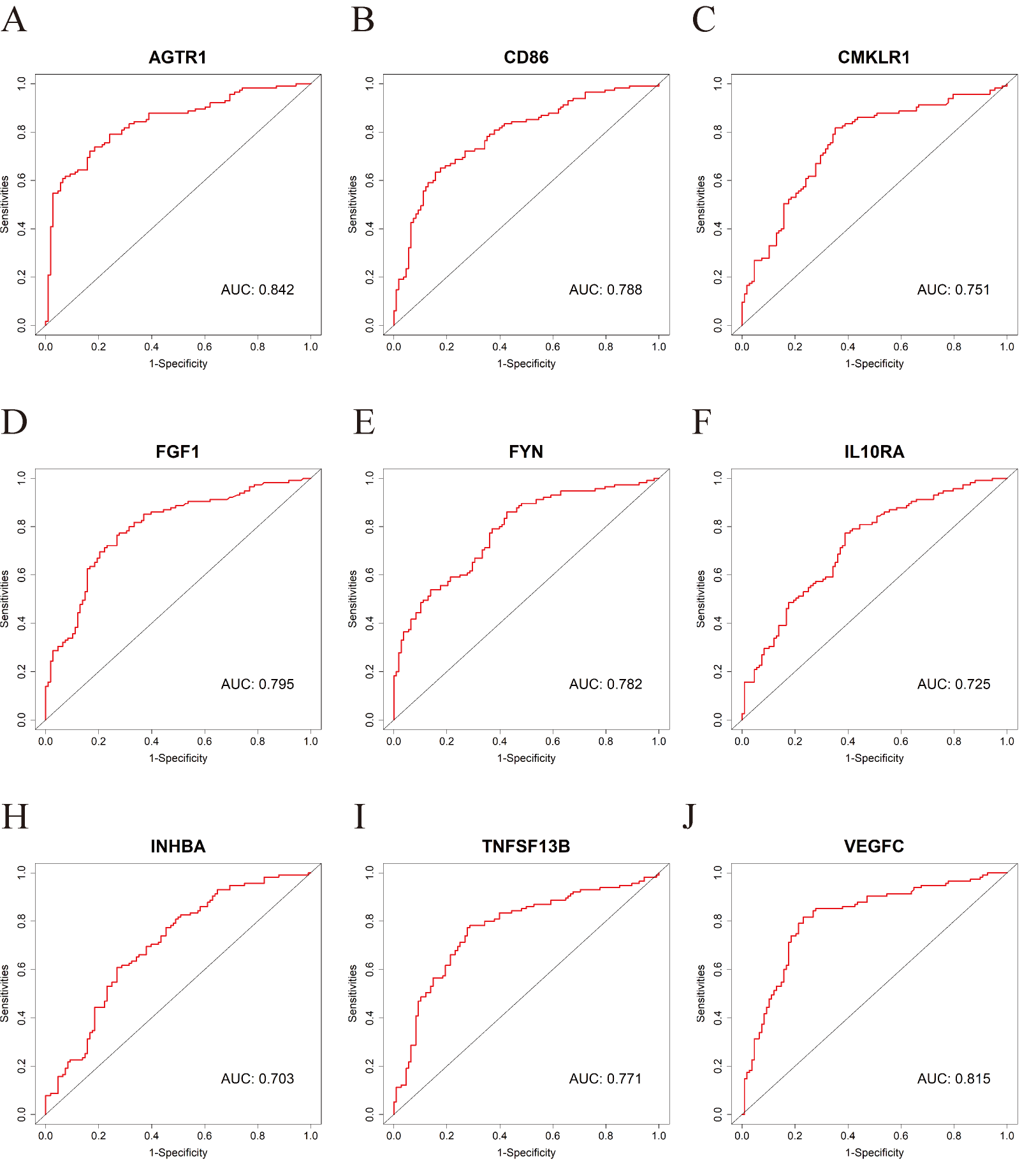

Supplement: Supplementary file 1 — Supplementary Material 1 [file 41598_2025_18589_MOESM1_ESM.zip › Figure 10.bmp]

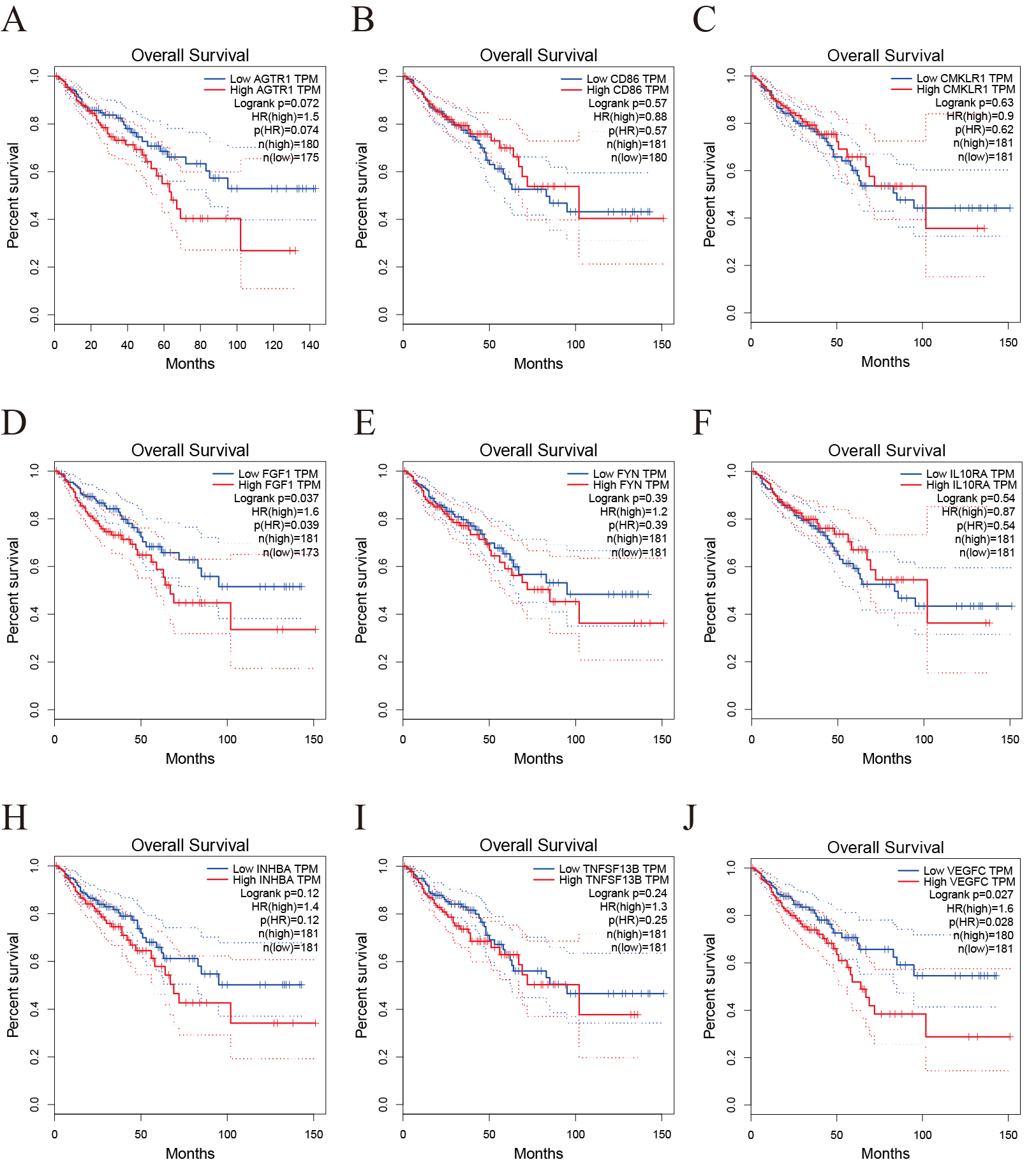

Supplement: Supplementary file 1 — Supplementary Material 1 [file 41598_2025_18589_MOESM1_ESM.zip › Figure 11.bmp]

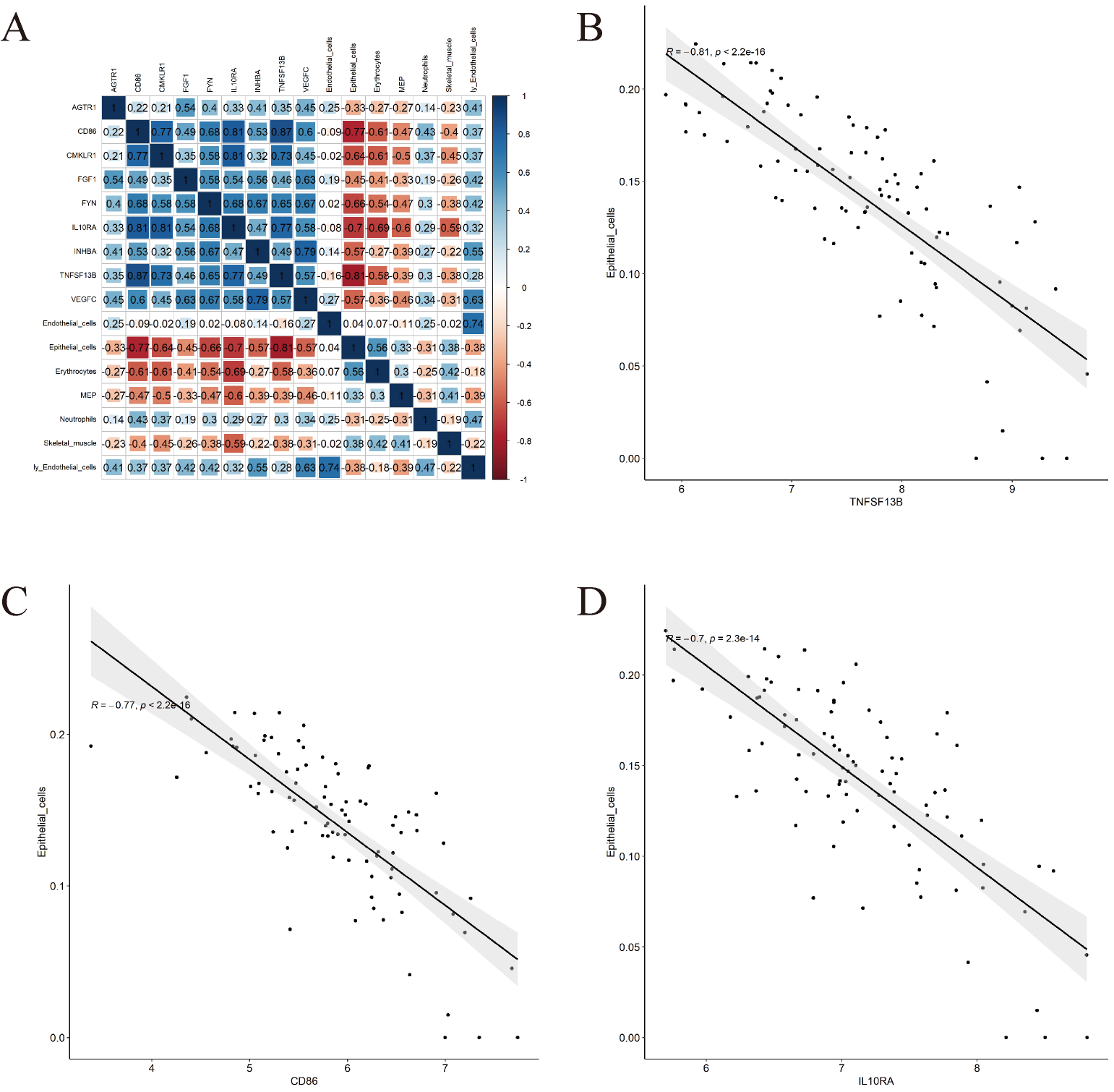

Supplement: Supplementary file 1 — Supplementary Material 1 [file 41598_2025_18589_MOESM1_ESM.zip › Figure 12.bmp]
